# Extrapulmonary tuberculosıs: an old but resurgent problem

**DOI:** 10.1186/s13244-022-01172-0

**Published:** 2022-03-07

**Authors:** Ali H. Baykan, Hakan S. Sayiner, Elcin Aydin, Mustafa Koc, Ibrahim Inan, Sukru M. Erturk

**Affiliations:** 1 Department of Radiology, Faculty of Medicine, Adiyaman University, Yunus Emre Mahallesi 1164 Sokak No:13, 02200 Merkez, Adıyaman Turkey; 2 Department of Infectious Diseases and Clinical Microbiology, Faculty of Medicine, Adiyaman University, Adiyaman, Turkey; 3 Department of Radiology, Izmir Tepecik Training and Research Hospital, Izmir, Turkey; 4 Department of Radiology, Faculty of Medicine, Firat University, Elazig, Turkey; 5 Department of Radiology,, King’s College Hospital London, Dubai, United Arab Emirates; 6 Department of Radiology, Faculty of Medicine, Istanbul University, Istanbul, Turkey

**Keywords:** Tuberculosis, Extrapulmonary tuberculosis, Magnetic resonance imaging, Computed tomography, X-ray

## Abstract

Tuberculosis (TB) primarily affects the lungs, but some of its most devastating clinical consequences arise because of its ability to spread from the lungs to other organs. Extrapulmonary TB (EPTB) constitutes 15–20% of all TB cases. Imaging findings are not always specific and can mimic many diseases; therefore, EPTB should be considered in the differential diagnosis, particularly in patients with immune system disorders (AIDS, patients receiving chemotherapy, etc.) and those in other high-risk groups including people with diabetes. The bacterium's passage to the regional lymph nodes is essential for developing a protective T-cell-mediated immune response, but the bacterium can spread hematologically and via the lymphatic system, leading to extrapulmonary involvement. Diagnosis of EPTB in high-risk patients is made based on suspected clinical and radiological findings, but further positive culture and histopathological confirmation may be required in some instances. Radiological evaluations are critical for diagnosis and crucial in planning the treatment and follow-up. This paper aims to review the typical and atypical imaging features and the differential diagnosis of EPTB.

## Key points

- EPTB is not an uncommon type of TB, but it is difficult to recognize.
- EPTB usually occurs with the lympho-hematogenous spread of primary infection (during or later) to extrapulmonary organs but can sometimes develop without pulmonary involvement.
- Radiological evaluations are critical for diagnosis and follow-up.

## Background

Tuberculosis (TB) is one of the infectious diseases with the highest preventable morbidity and mortality rates globally [1]. According to the World Health Organization (global TB report 2021), an estimated 10 million people fall ill with TB, and 1.5 million die from TB every year [2]. High-risk groups include immunosuppressed individuals, malnourished people, people of low socioeconomic status, prisoners, alcoholics, children, the elderly, the homeless, nursing home residents, those living in TB-endemic areas, and healthcare workers [3]. One in every five TB cases presents with extrapulmonary TB (EPTB) [3]. The tuberculin skin test, serum interferon-gamma release, polymerase chain reaction, adenosine deaminase assays, and imaging modalities are used to diagnose EPTB, although biopsies and culture studies remain the gold standard [3–5]. EPTB can occur almost anywhere in the body, most commonly in the lymph nodes (50%), the pleura (18%), the genitourinary system (13%), bones and joints (6%), the gastrointestinal system (6%), the central nervous system (CNS) (3%) and the spine (3%) [1].

EPTB shows various clinical and radiological features depending on the organ it affects, which can often mimic other diseases [6]. Therefore, early diagnosis and treatment are essential. Radiological modalities can be used separately or together, depending on the affected organ and the extent of the involvement. For example, X-rays and computed tomography (CT) are conducted if there is bone involvement, ultrasound (US), CT and magnetic resonance imaging (MRI) in cases of possible abdominal TB, the US in the presence of a superficial lesion such as lymphadenitis, mammography, and the US if mastitis has developed, and MRI if the CNS is affected. MRI helps show whether deep tissues are affected by TB in the presence of musculoskeletal involvement. Additionally, positron emission tomography (PET)-CT can evaluate the extent of skeletal TB and monitor the response to treatment [3, 7]. This review emphasizes radiology’s role in diagnosing EPTB by presenting examples of TB that involve different parts of the body.

## Pathophysiology

TB is a chronic bacterial infection caused by mycobacteria called the *Mycobacterium tuberculosis* complex (*Mycobacterium africanum*, *Mycobacterium microti*, *Mycobacterium tuberculosis*, and *Mycobacterium bovis*). It is characterized by the presence of caseous granulomas in infected tissues. *M. tuberculosis* is the cause of the disease in 97–99% of cases. TB is an aerobic, acid-fast, non-motile, non-encapsulated, non-spore bacillus discovered by Robert Koch in 1882 [8]. It can involve almost all organs but is most frequently found (80–85%) in the lungs [1, 4, 9].

The cell-wall structure of mycobacteria is different from other bacteria; they have an intracellular lifestyle and chronic infection properties and are resistant to chemical structures including acid and alkaline environments, antibiotics, disinfectants, enzymes, and free radicals. The cell structure prevents phagolysosomal fusion formation within the macrophage, which plays a role in immunity [10].

The disease is most commonly transmitted by entering *M. tuberculosis* into the lungs via droplets from person to person or transferring *M. bovis* into the gastrointestinal tract by consuming non-pasteurized dairy products. Although rare, cases involving congenital transmission, sexual transmission, vaccination, and therapeutic installation have also been reported [11].

The TB bacillus spreads to the lung alveoli via droplets, phagocytoses by alveolar macrophages, and multiplies within macrophages. Alveolar macrophages differentiate from macrophages to histiocytes by interacting with T lymphocytes. Epithelioid histiocytes and lymphocytes gather in small clusters and form granulomas to restrict the disease [12, 13]. This granuloma (with a central area of caseous necrosis) containing bacteria is called primary lesion or Ghon focus [12, 14]. If this granuloma breaks up, aerosols are formed that can infect other people via expectoration. [12].

**Fig. 1:**
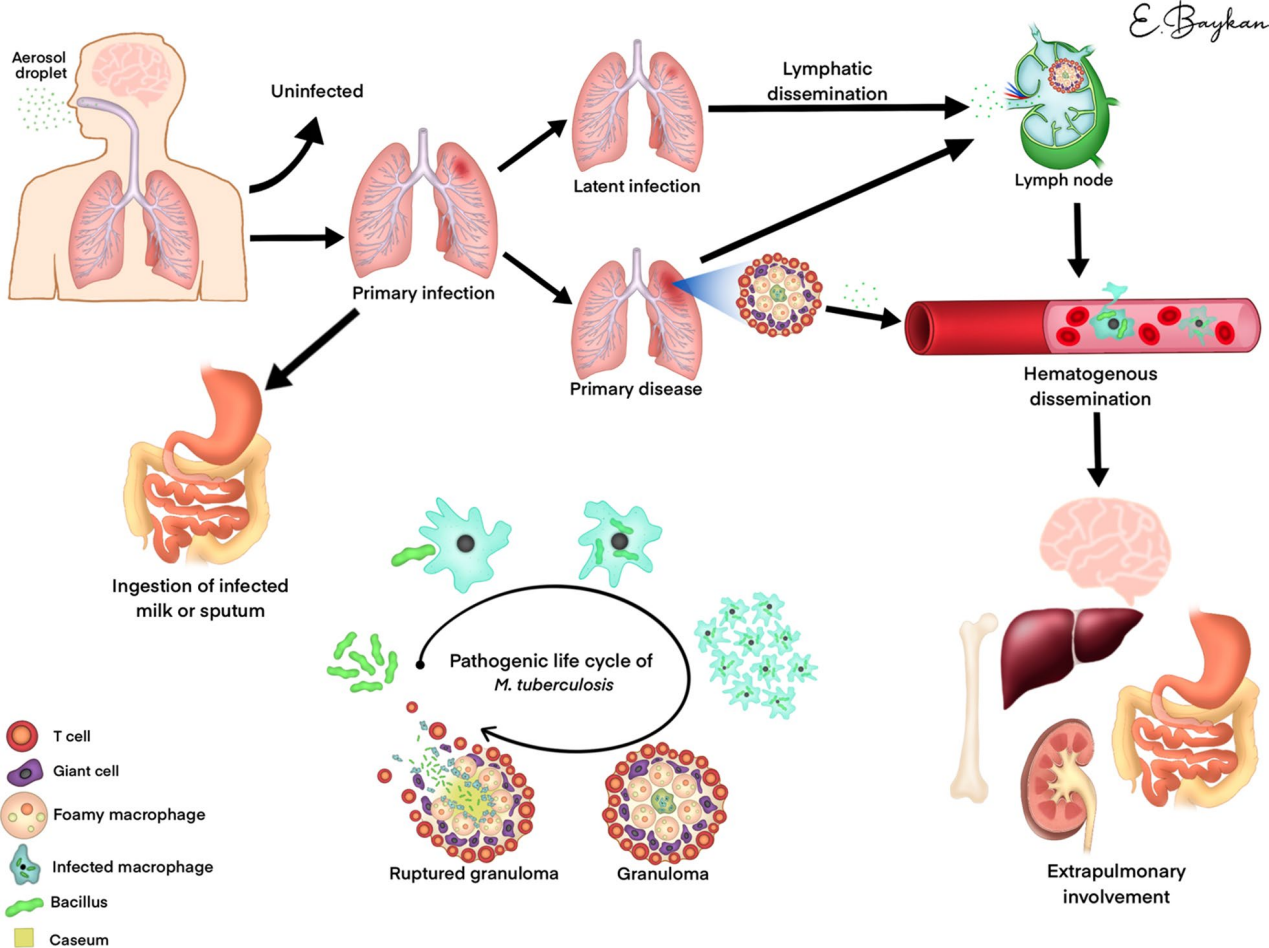
Pathogenic life cycle and extrapulmonary dissemination of M. tuberculosis

Bacillus growth in macrophages and alveolar spaces are followed by new monocyte migration from the blood to the lesion area and the migration of macrophage-mediated bacilli to neighboring lymph nodes. The bacilli can then spread from lymph nodes to the subclavian vein and throughout the body via the hematogenous route. Using the lymphohematogenous route, the bacillus creates new infection foci until an immune response is triggered [9]. Thus, different organ involvements occur. Extrapulmonary involvement may present with or without pulmonary infection [15], and EPTB can affect any organ or tissue except for the hair, nails, and teeth. [16]. The pathogenic life cycle and extrapulmonary spread of *M. tuberculosis* are summarized in Fig. 1.

## Involvement of the body systems

### Nervous system

CNS involvement has been found in 5–10% of patients with TB and 20% of those with AIDS-related TB [17]. It is one of the most significant causes of morbidity and mortality in TB-endemic areas. CNS-TB is usually caused by hematogenous spread, often directly from an intracranial or extracranial focus [3, 18]. Clinical features are variable. However, a nonspecific headache, neck stiffness, fever, vomiting, radiculopathy, and even coma can appear in most cases [19]. The clinical and radiological manifestations of CNS-TB can mimic many inflammatory, infectious, and tumoral diseases.

Cranial involvement of TB may present as extra-axial (tuberculous leptomeningitis and tuberculous pachymeningitis), intra-axial (tuberculoma, focal cerebritis, tuberculous abscess, tuberculous rhombencephalitis), or tuberculous encephalopathy [3].

#### Tuberculous leptomeningitis

**Fig. 2:**
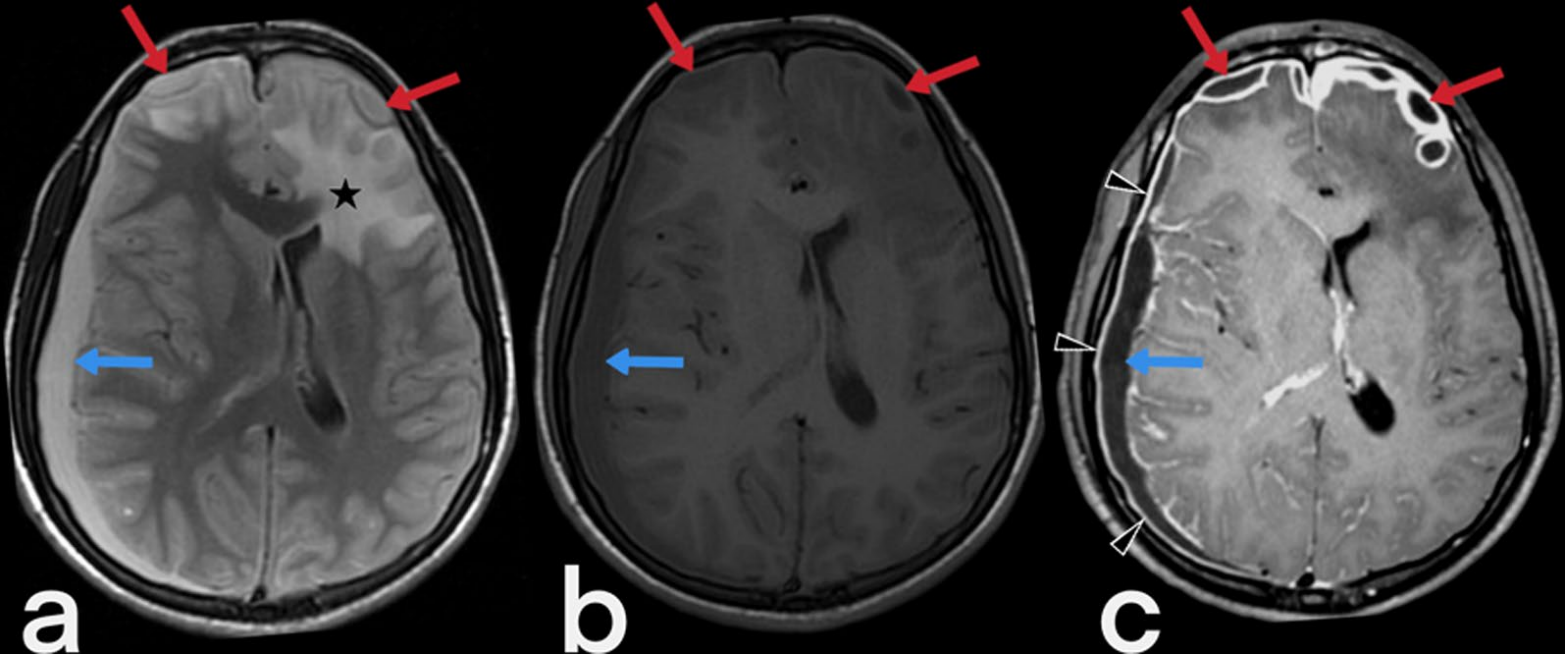
Axial FLAIR (**a**), pre-contrast (**b**) axial T1 weighted, and post-contrast (**c**) axial T1 weighted MR images of a 21-year-old male. Axial FLAIR image shows high signal intensity caused by edema and inflammation in the left frontal lobe (star). In both anterior frontal regions (red arrows) and right parietal area (blue arrows) are seen subdural abscesses. In addition, MR images demonstrate meningeal enhancement and thickening (arrowheads)

Leptomeningitis is the most common CNS-TB finding in children and adolescents and develops due to the hematogenous spread of *M. tuberculosis*. The presence of exudate, which develops in the subarachnoid space at the base of the brain, is specific to tuberculous leptomeningitis and can be seen with CT and contrast-enhanced MRI in particular (Fig. 2). The most common location is the interpeduncular fossa. In the differential diagnosis, infective meningitis and inflammatory diseases such as rheumatoid arthritis, sarcoidosis, and carcinomatous meningitis should also be considered [20].

Possible complications of tuberculous leptomeningitis include progressive hydrocephalus, vasculitis, infarction, and cranial neuropathies [3]. The most common complication is communicating hydrocephalus, which is caused by the obstruction of cerebrospinal fluid into the basal cisternae. In some cases, non-communicating hydrocephalus caused by a tuberculoma or tuberculous abscess may also be seen [17]. Ischemic infarction may also develop as a result of cerebral arteritis. Hemorrhagic infarction caused by dural venous sinus thrombosis is another complication of tuberculous leptomeningitis. Cranial nerves 2, 3, 4, and 7 may be affected by vascular ischemia or nerve entrapment, and the diagnosis is made with nerve thickening and enhancement in MRI [21].

#### Tuberculous abscess

Tuberculous abscesses are rarely seen. A tuberculous abscess is either solitary or multiple lesions and is usually multiloculated with liquefaction necrosis in the central area. CT and MRI are visualized as a mass with a cystic center, surrounding edema, and a contrast-enhanced wall [21]. A tuberculous abscess is different from a tuberculoma, which presents with caseous necrosis and liquefaction in the center.

#### Tuberculoma

The center of a tuberculoma contains necrotic caseous material surrounded by a capsule comprising fibroblasts, epithelioid cells, Langhans giant cells, and lymphocytes. Lesions may be solitary, multiple, or miliary, and the frontal and parietal lobes are often involved. Ring-like enhancement is observed on CT, and the typical finding of a tuberculoma is a central nidus of calcification with surrounding ring-like enhancement, known as the target sign. The MRI appearance of tuberculomas varies depending on whether or not there is caseation [17, 22].

**Fig. 3:**
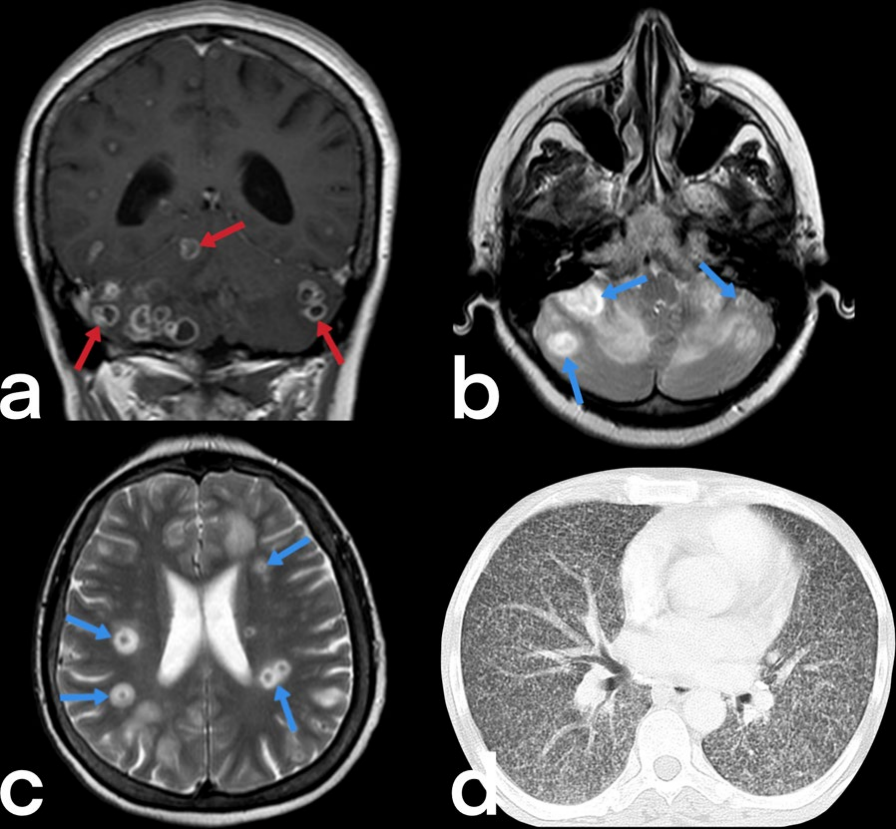
Radiologic images of a 38-year-old-female who has a diagnosis of miliary TB. Multiple tuberculomas (red arrows) show peripheral rim-like enhancement on post-contrast T1 weighted image in the cerebellum (**a**). On T2-weighted images (**b,** **c**) demonstrate focal lesions (tuberculomas) with central isointense signal and peripheral hyperintensity in gray matter (blue arrows). Multiple nodules secondary to miliary TB are seen (**d**)

**Fig. 4:**
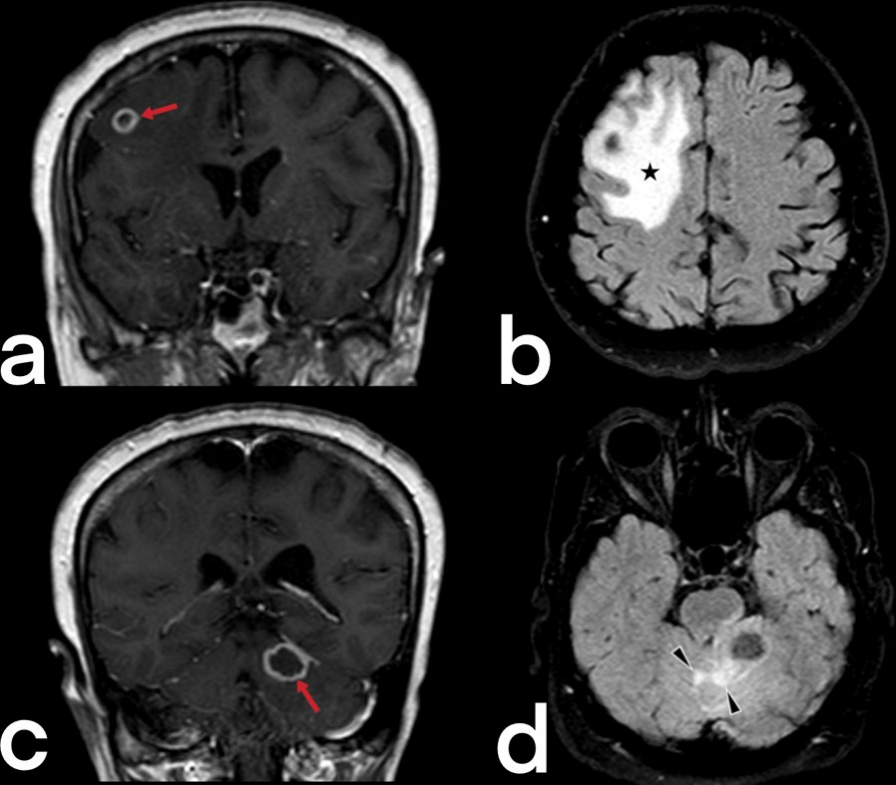
MR images of a 63-year-old female with central nervous system TB. Focal lesions (arrows) with rim-like enhancement are seen (tuberculoma) on contrast-enhanced coronal T1 weighted images (**a, c**). Perilesional increased signals (asterisk and arrowheads) due to vasogenic edema are noted on axial FLAIR images (**b, d**)

Non-caseating tuberculomas are solid lesions that are non-necrotic, are located in subcortical white matter, are hypointense on T1-weighted images, hyperintense on T2-weighted images, show post-contrast homogeneous enhancement, and are surrounded by vasogenic edema [17]. Caseating tuberculomas are seen in two forms as solid caseating tuberculomas (early stage) and tuberculomas with central liquefaction (late-stage). Solid caseating tuberculomas appear hypointense or isointense on T1-weighted images and hypointense on T2-weighted images caused by paramagnetic radicals that develop due to the inflammation and the capsule being stained in the form of a ring in contrast-enhanced sections (Figs. 3, 4c) [17]. In contrast, the tuberculomas with central liquefaction present centrally hyperintense signal and peripheral hypointense rim (capsule) on T2-weighted images (Fig. 4b) [20, 21]. Neurocysticercosis, fungal granulomas, pyogenic abscesses, tumors such as lymphomas, gliomas, and metastases are included in the differential diagnosis of tuberculomas [22].

#### Spinal cord TB

**Fig. 5:**
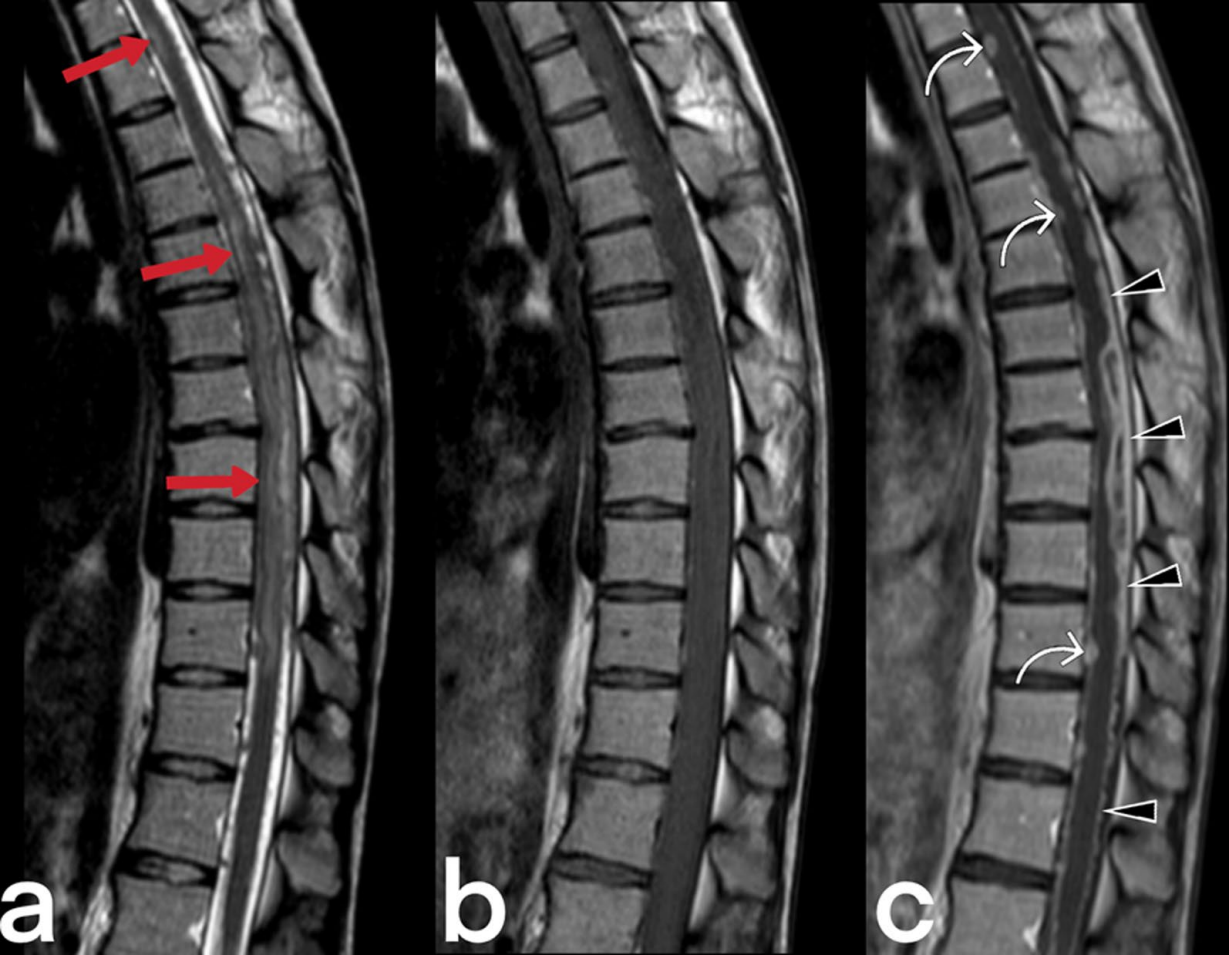
MR images of thoracic vertebrae of a 23-year-old female. Sagittal T2-weighted image reveals a heterogeneous signal increase in the spinal cord (red arrows) (**a**). Precontrast (**b**) and postcontrast (**c**) T1 weighted images of the same patient show linear (arrowheads) and nodular (curved arrow) dural enhancement and tuberculous abscess formations in the posterior epidural space

**Fig. 6:**
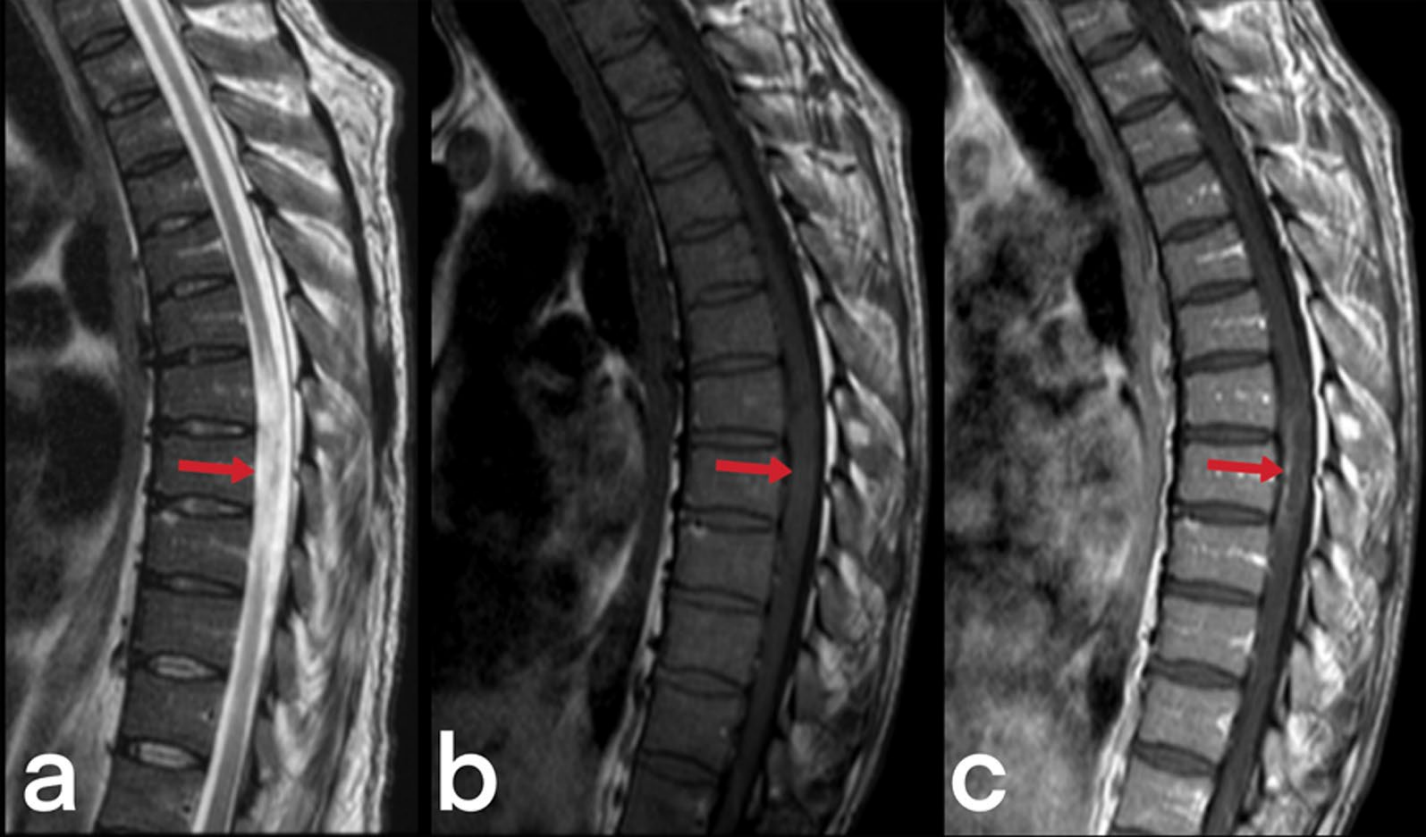
MR images of a 56-year-old male with the diagnosis of tuberculous myelitis. Sagittal T2 weighted image (**a**) shows a heterogeneous increased signal on the spinal cord (arrow). Pre-contrast (**b**) and post-contrast (**c**) T1 weighted images reveal linear contrast enhancement of the same region (arrows)

**Fig. 7:**
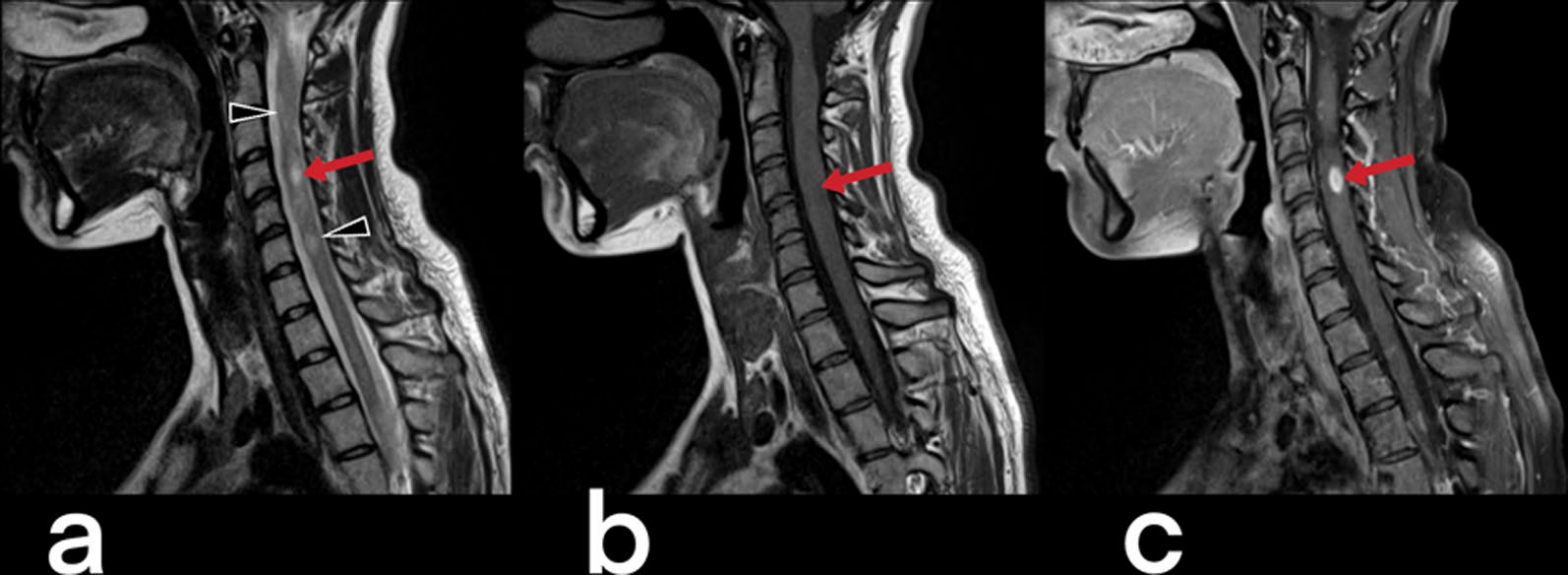
MR images of a 17-year-old female with the diagnosis of cervical tuberculous myelitis. Sagittal T2 weighted image (**a**) shows a heterogeneous increased signal (edema, inflammation) on the spinal cord (arrowheads) and focus (arrow). The cervical spinal cord is observed as an enlarged diffuse hypointense area on pre-contrast T1-weighted image (**b**). Post-contrast (**c**) T1 weighted image reveals diffuse heterogeneous and focal (arrow) contrast enhancement of the same region. The focus is a tuberculoma (arrows)

Spinal cord TB can be observed in cases of intramedullary myelitis, spinal meningitis, and arachnoiditis. It develops because of the direct spread of tuberculous spondylitis from the vicinity or hematogenous spread from the primary focus [23]. Spinal edema and segmental infarcts may develop due to infective thrombosis and vasculitis in the spinal arteries [23]. An increase in cord signal is observed in T2-weighted MRI images caused by thick, irregular meningeal involvement (Fig. 5), spinal cord and root inflammation, myelitis (Fig. 6), edema, and infarction [23, 24]. Syringomyelia caused by chronic arachnoiditis can also be detected in the later stages [23]. Extreme rarely (only 2 out of 1000 cases of central nervous system TB), it may present as intramedullary spinal tuberculoma (Fig. 7) [25]. Other spinal cord lesions (for example, myelitis, astrocytic glioma, ependymocytoma, and hemangioblastoma) should be considered in the differential diagnosis [25, 26].

### Head and neck

#### Tuberculosis of the larynx

**Fig. 8:**
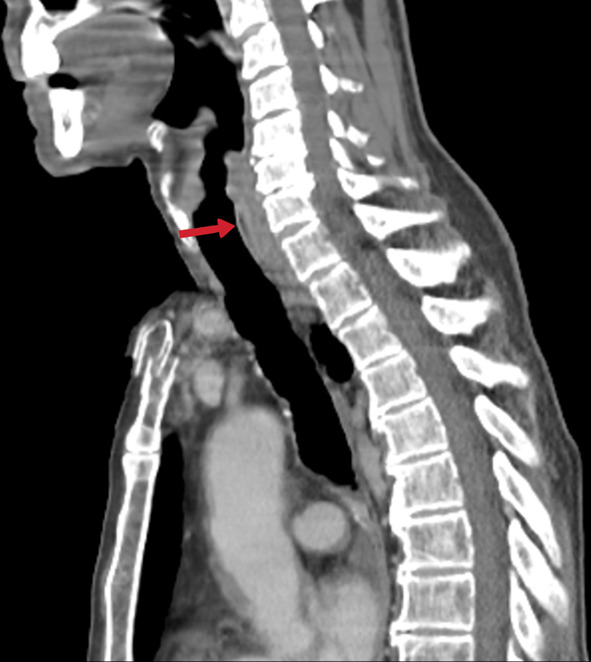
A 68-year-old-male. Sagittal reformatted contrast-enhanced CT image reveals an increased thickness of the larynx's posterior wall (arrow). Sternum fracture is also noted in the manubrium

Laryngeal TB develops in 1 out of every 100 cases with lung TB, and almost all cases with laryngeal TB have primary lung TB. This result is explained by the direct contact of bronchogenic secretions with the laryngeal mucosa. Rarely, laryngeal tuberculosis may develop without pulmonary involvement [27]. In a patient with pulmonary TB, the lesion in the larynx should first suggest laryngeal TB. Its symptoms are hoarseness, pain, and odynophagia. Vocal folds, pre-epiglottic and paraglottic areas show soft tissue thickening and inflammatory changes (Fig. 8) [20].

#### Tuberculous lymphadenitis

**Fig. 9:**
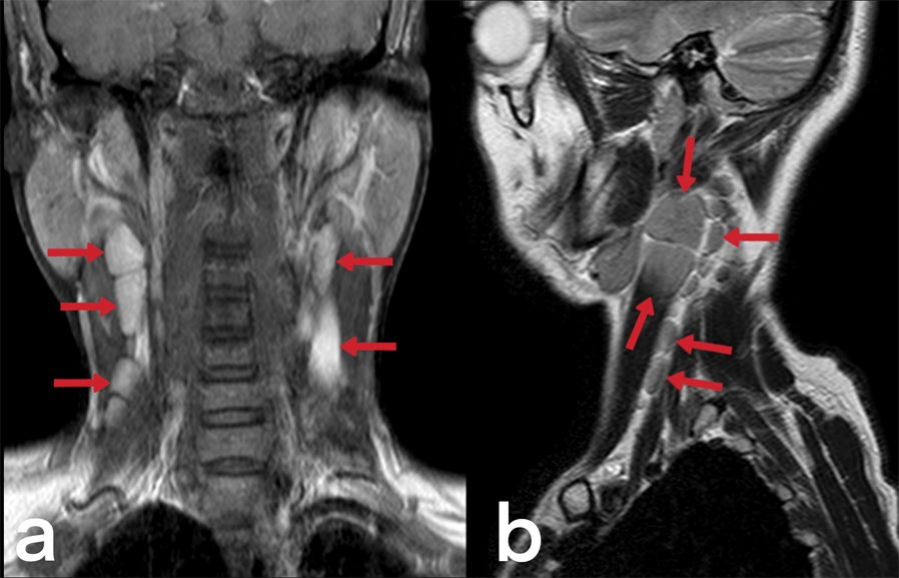
Contrast-enhanced T1 weighted coronal (**a**) and sagittal T2 weighted (**b**) MR images of a 39-year-old female demonstrate multiple lymphadenopathies in the bilateral cervical chains. The patient was diagnosed with tuberculous lymphadenitis

**Fig. 10:**
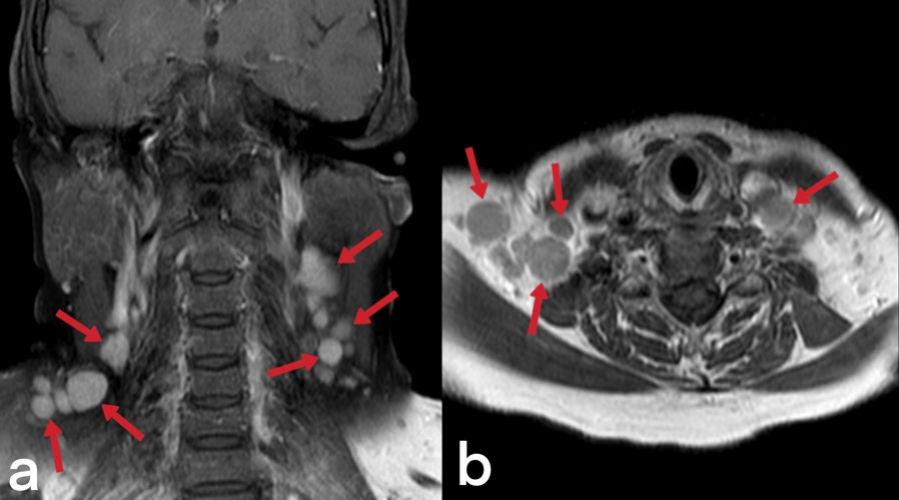
Contrast-enhanced T1 weighted coronal (**a**) and axial T1 weighted (**b**) MR images of a 44-year-old female demonstrate multiple lymphadenopathies in the bilateral cervical chains and right supraclavicular region. Multiple intraabdominal lymphadenopathies are also noted (not shown)

**Fig. 11:**
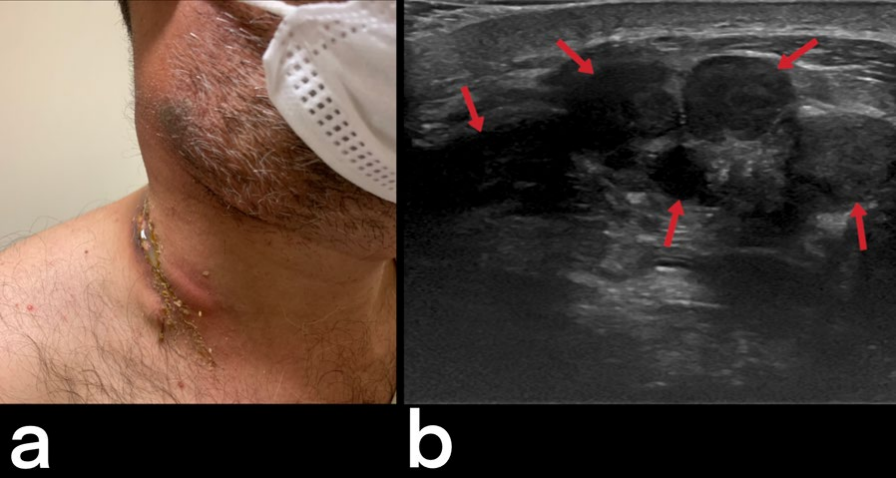
A 32-year-old male. Painless, discrete, freely mobile, fistulized lumps in the neck are grown progressively (**a**). Ultrasound showed multiple necrotic lymph nodes (**b**). Scrofula of the neck

Tuberculous cervical lymphadenitis is also known as scrofula and is the most common form of EPTB seen in endemic populations [3]. Cervical lymphadenitis may develop during primary lung infection or via lymphatic dissemination from the mediastinal lymph nodes after the latent period [4, 23]. Although lymph nodes initially appear homogeneous (Figs. 9, 10), they may later be observed as nodular formations that show low CT density due to central necrosis. Calcification can also be seen in lymph nodes in the later stages [23, 28]. Ultrasound findings are variable. While the first finding is a round-shaped nodal enlargement in the early stage, abscess, necrosis, and fistula formation are more common in the late period (Fig. 11) [29]. Localizations of tuberculous lymphadenitis include cervical (63%), mediastinal (27%), and axillary (8–10%) nodes. Most cases present with unilateral cervical lymphadenopathy [3]. The differential diagnosis considers the metastatic necrotic lymph nodes of papillary thyroid carcinoma and squamous cell cancer [23].

#### Other regions

**Fig. 12:**
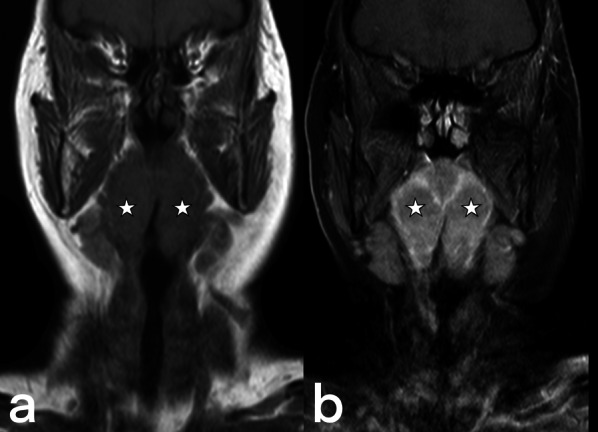
A 50-year-old female. Coronal pre-contrast (**a**) and post-contrast (**b**) T1 weighted images reveal enlarged palatine tonsils, which show peripheral enhancement (stars)

**Fig. 13:**
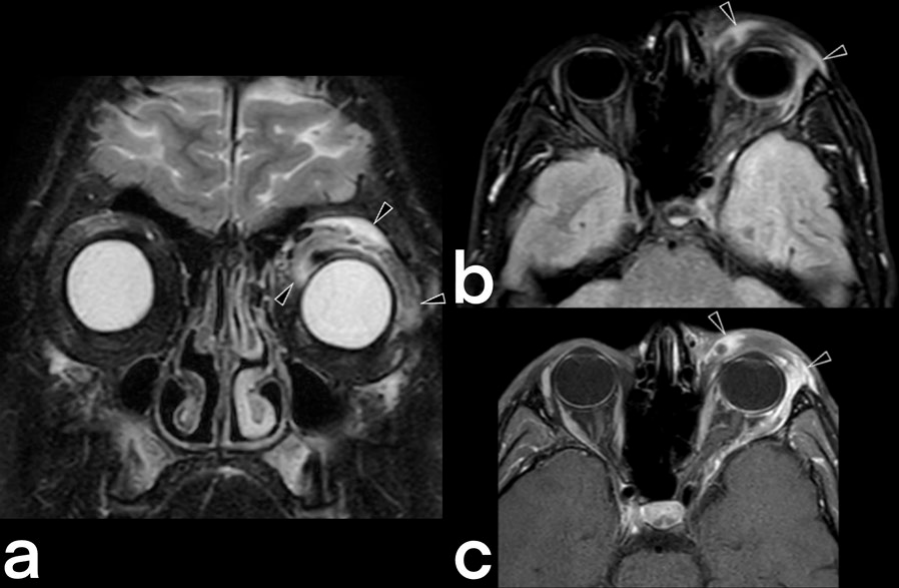
A 19-year-old male patient. T2 weighted coronal (**a**), axial FLAIR (**b**), and T1 weighted post-contrast axial (**c**) images show periorbital involvement (arrowheads)

**Fig. 14:**
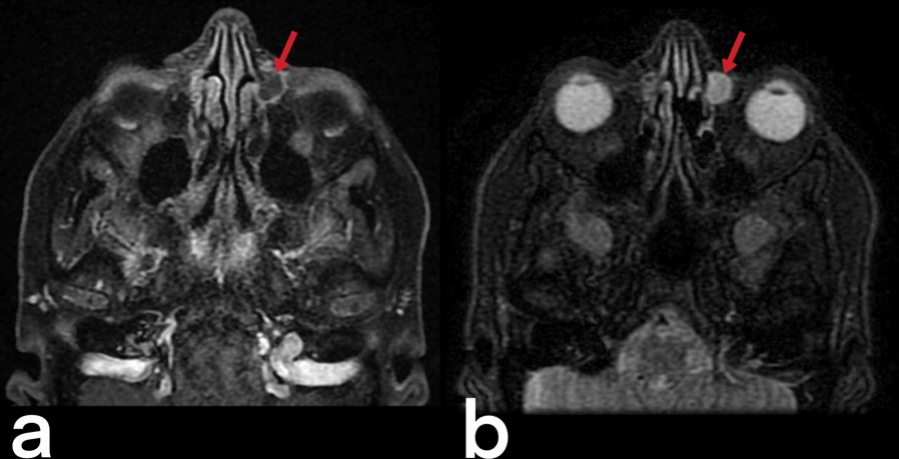
A 28-year-old female patient. T1 weighted post-contrast image (**a**) and T2 weighted image (**b**) with a tuberculous abscess in the left lacrimal sac (arrows)

**Fig. 15:**
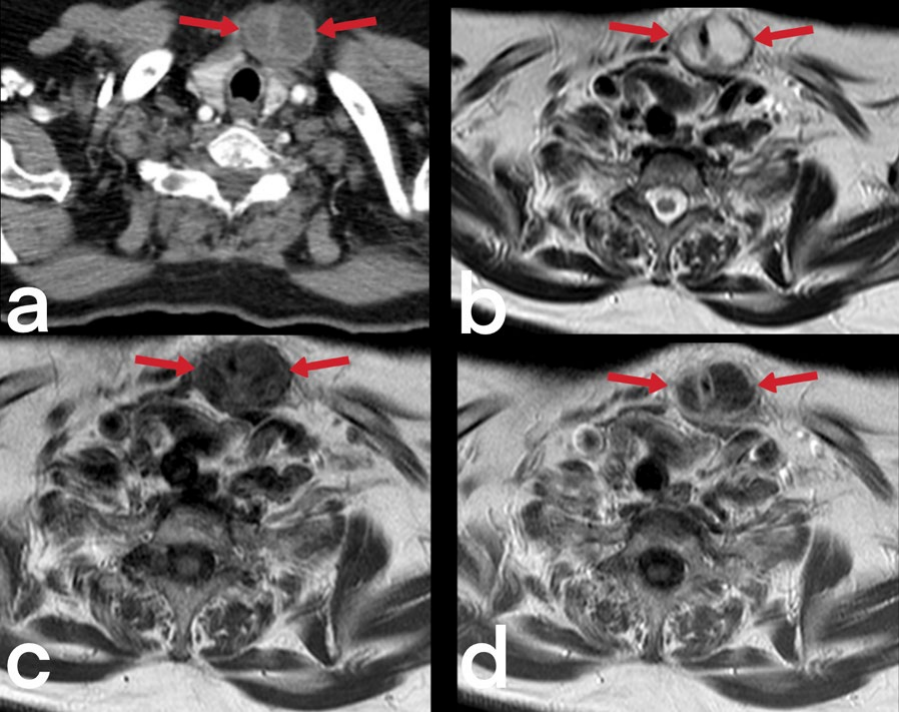
Axial contrast-enhanced CT (**a**), axial T2 weighted (**b**), pre (**c**), and post-contrast T1 (**d**) weighted images of a 69-year-old female. Biloculated abscess formation is seen in the anterior left paramedian region of the neck, which shows peripheral enhancement

The areas less frequently involved in the head and neck region include the temporal bone, pharynx, tonsil, sino-nasal cavity, thyroid gland, skull base, lacrimal sac, bulbus oculi, and other regions of the neck [20]. Primary involvement is infrequent. It usually develops as a result of hematogenous dissemination (e.g., thyroid) after the lung infection, contact with the infected sputum, or saliva (e.g., eye globe) or through the ingestion of unpasteurized milk (e.g., tonsil, pharynx) [27, 30, 31]. It is generally observed as nonspecific inflammatory soft tissue thickening (Figs. 12, 13) and abscess (Figs. 14, 15). In advanced cases, soft tissue masses and bone erosions can also be detected [20].

In tuberculous otomastoiditis, increased soft tissue density is observed in the tympanic cavity on CT in the early stages, while destruction in the middle ear structures and retroauricular/epidural abscesses can be seen in the later stages. The differential diagnosis includes pyogenic or fungal infection, sarcoidosis, cholesteatoma, and Wegener's granulomatosis [6].

Chorioretinitis and uveitis are common in ocular TB. Imaging findings show a unilateral choroidal mass, with melanoma, metastasis, hemangioma, sarcoidosis, and systemic mycosis considered in the differential diagnosis [6].

### Mediastinum

**Fig. 16:**
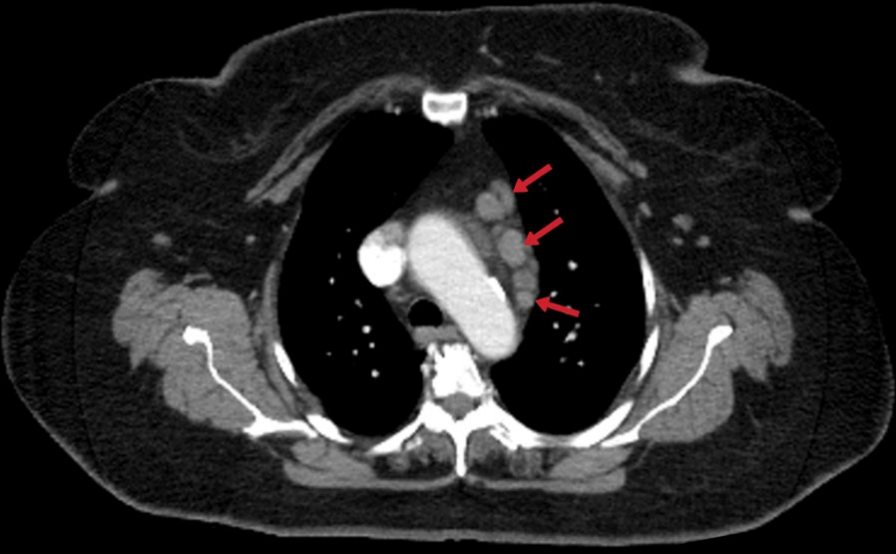
Contrast-enhanced thorax CT image of a 70-year-old female. Multiple mediastinal lymphadenopathies are noted in the prevascular region (red arrows)

**Fig. 17:**
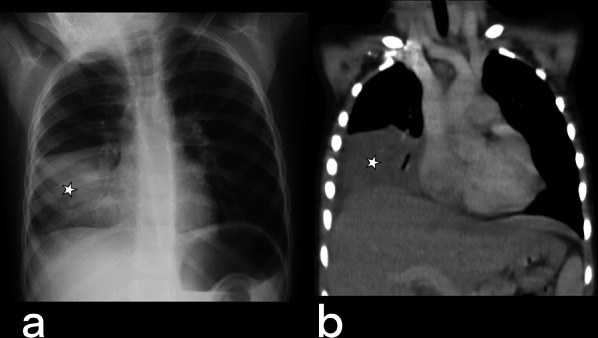
The chest X-ray (**a**) and coronal reformatted CT (**b**) image without contrast of a 4-year-old girl demonstrate unilateral pleural effusion in the right side (stars). Tuberculous pleurisy

**Fig. 18:**
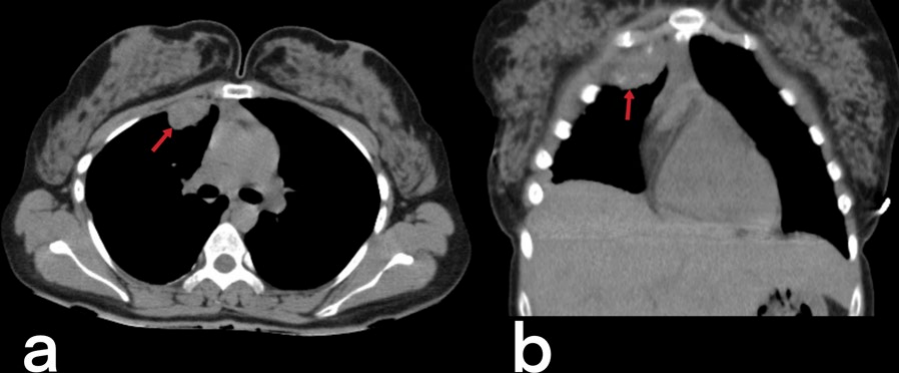
Transverse (**a**) and coronal reformatted (**b**) CT images without contrast of a 24-year-old female. A mass-like focal pleural lesion in the right side containing a few coarse calcifications is noted. The patient is diagnosed with TB on histopathologic assessment after surgical removal of the mass

Mediastinal lymphadenitis is more common in children with TB than in adults. Tuberculous lymphadenitis is usually observed unilaterally in the right hilar and paratracheal area. In CT, necrotic lymph nodes larger than 2 cm indicate the presence of active disease (Fig. 16). Calcified lymph nodes and Ghon focus form the Ranke complex [20]. Pleural effusions or empyema can be seen in cases of TB (Fig. 17), and tuberculous empyema can sometimes mimic a thoracic wall mass (Fig. 18) [5].

### Cardiac

**Fig. 19:**
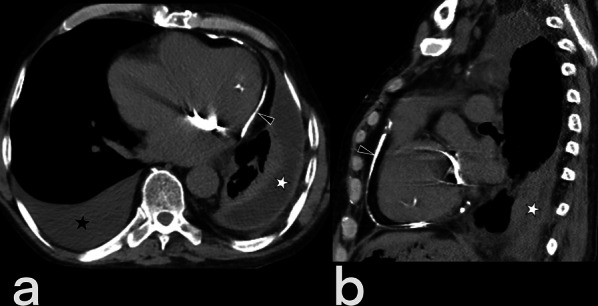
Transverse (**a**) and sagittal reformatted (**b**) CT images without intravenous contrast of a 55-year-old male. Pericardial thickening with linear calcification (arrowheads), right-sided pleural effusion (black asterisk), and infected fluid collection (white asterisk) in the left pleural cavity are seen. Mediastinal and pericardial tuberculous

Cardiac TB is very rare (only 0.5% of cases of extrapulmonary tuberculosis), although pericardial involvement is seen more frequently than myocardial involvement [20]. Involvement of the heart by TB can appear by hematogenous seeding from infected lung, direct spread from the adjacent focus or, via a lymphatic feed from mediastinal lymph nodes [32]. In tuberculous pericarditis, pericardial thickening (> 3 mm), irregular contours, and mediastinal lymphadenopathy are observed. An inferior vena cava diameter of > 3 cm, bilateral pleural effusion, intraventricular septum deformities, pericardial effusion, and localized pericardial calcification can also be observed (Fig. 19). Myocardial involvement is asymptomatic but can be detected postmortem [3, 20].

### Breast

Breast TB is a rare form of EPTB that affects multiparous and lactating women between 20 and 40 years old. While the spread of infection causes the primary form through abrasions in the skin of the breast or through cracks in the nipple, the secondary form can be caused by retrograde spread from infected axillary lymph nodes or by direct spread from adjacent tissue [7].

The patient's general condition is good, and systemic findings are not generally observed. The clinical presentation is nonspecific and variable, so it is difficult to distinguish clinically between granulomatous mastitis and breast cancer. However, in most cases, a painless palpable mass is often found in the breast's upper outer quadrant or central area [7].

**Fig. 20:**
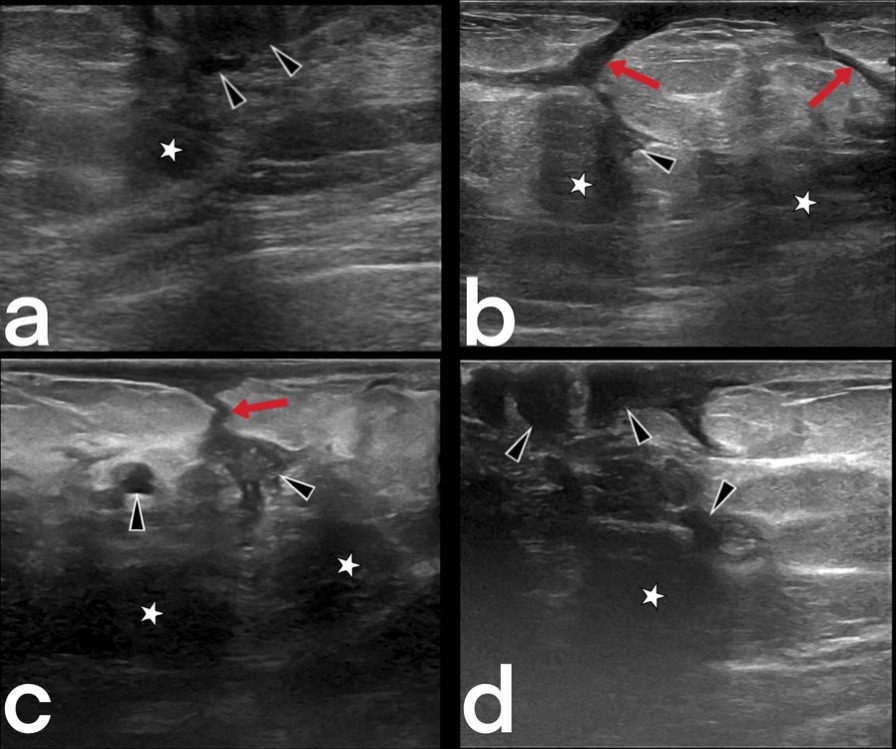
Ultrasound images from four different female patients (**a**–**d**). Abscesses (arrowheads), sinus tracts (arrows), and hypoechoic parenchymal edema areas (stars)

**Fig. 21:**
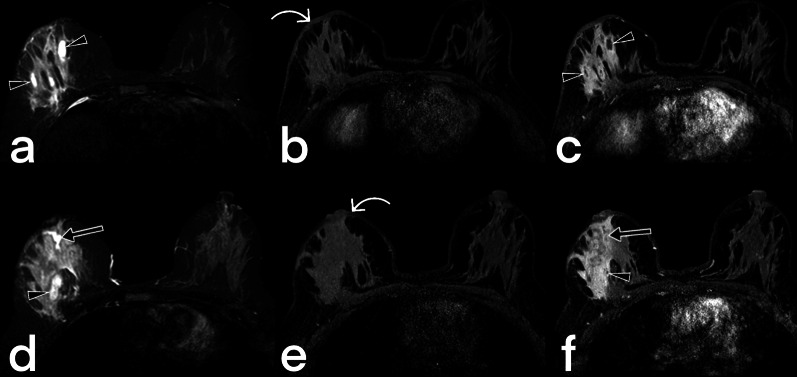
MR images of a 41-year-old female. Axial T2 weighted (**a**, **d**), pre-contrast axial T1 weighted (**b**, **e**), and post-contrast axial T1 weighted (**c**, **f**) images reveal diffuse increased signal intensity, cystic lesions (arrowheads), ductal ectasia (arrow), and thickened skin (curved arrow) in the right breast. Diffuse-type breast TB

**Fig. 22:**
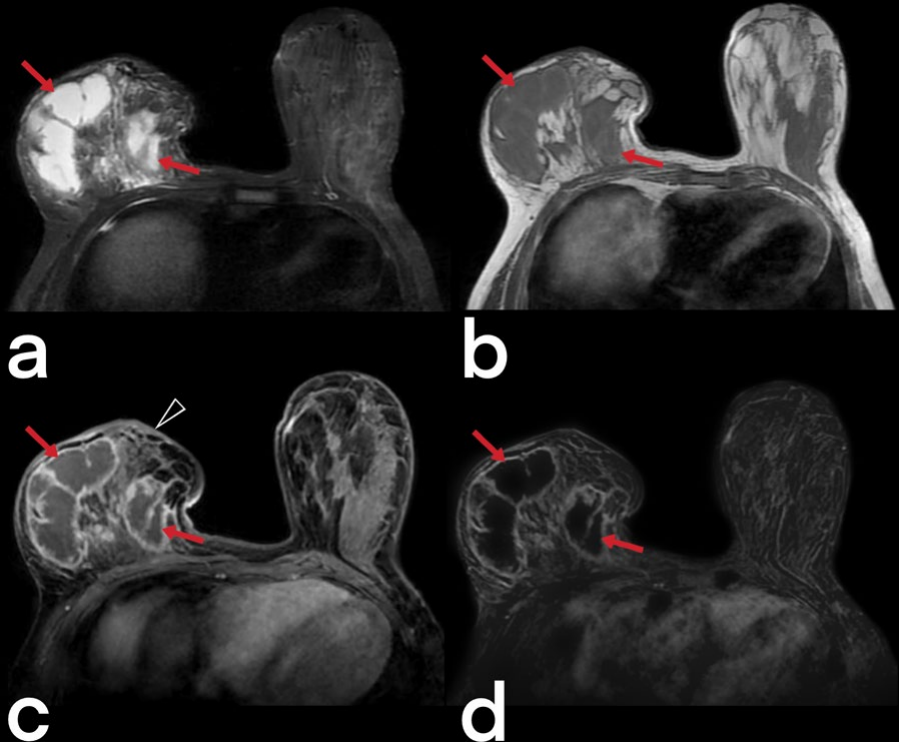
Breast MR images of a 39-year-old female. T2 weighted image (**a**), T1 weighted image (**b**) post-contrast (**c**), and subtracted image (**d**) demonstrate thickening of the skin (arrowhead) and multiple abscesses (arrows) with peripheral enhancement in the right breast

Breast tuberculosis is radiologically observed in three forms: nodular, diffuse, and sclerosing [33]. Because females of reproductive age are affected, US is usually the first-choice imaging modality. The disease may present as a circumscribed or indistinct mass. Additional findings such as abscesses, fistulas, sinus tracts, increased skin thickness/retraction can be observed, but suspicious calcifications are not generally expected (Fig. 20) [7, 34, 35]. MRI can show the extension of an abscess into extramammary areas and can also show the fistula tract's continuation into deep tissues (Fig. 21, 22) [7].

Histologically, breast TB is a form of granulomatous inflammation, so it is not easy to diagnose it pathologically. A core needle biopsy generally supports the diagnosis. Diagnosing breast TB has three essential pillars: clinical examination, radiological evaluation, and histopathological sampling [7].

### Abdomen

Abdominal TB can be developed as direct involvement (via ingestion of infected lung secretions, infected unpasteurized dairy products, or infected undercooked meats) or at the time/or after the primary lung infection via spreading the lymphatic/hematologically from a focus elsewhere in the body. TB can affect all abdominal organs and tissues [3, 4]. Clinical manifestations are nonspecific and depend on the organ involved, the extent of the affected site, and the duration of the disease (acute, chronic, or acute upon chronic). In general, clinical features may include fever (40–70%), pain (80–95%), weight loss (40–90%), ascites, diarrhea, anorexia, malaise, and bowel obstruction [36, 37].

#### Abdominal lymphadenopathy

**Fig. 23:**
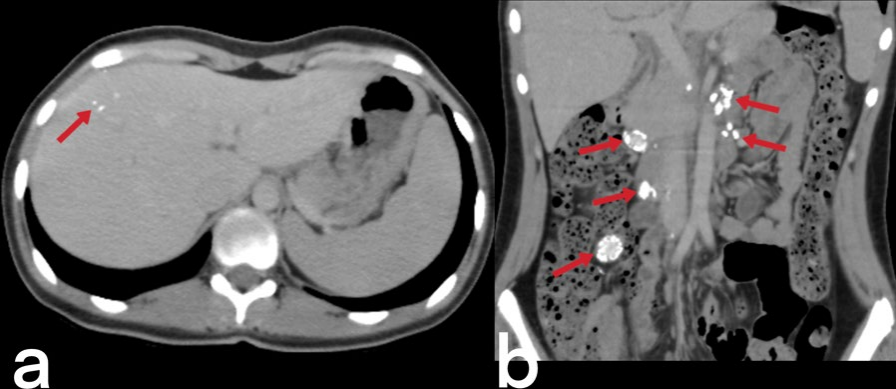
Transverse abdomen CT image (**a**) of a 17-year-old female with known TB. Coarse calcifications in the liver parenchyma suggestive of TB sequela are noted. Coronal reformatted CT image of the same patient (**b**) reveals multiple lymphadenopathies with peripheral calcifications

**Fig. 24:**
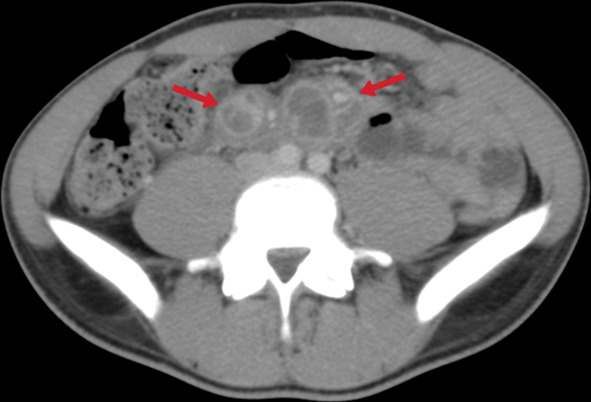
A 19-year-old male. Post-contrast CT image shows multiple mesenteric lymphadenopathies with peripheral enhancement

Abdominal lymphadenopathy is the most common abdominal TB involvement, seen in 55–66% of patients. CT is observed as group-forming, round-shaped, and centrally hypodense lymph nodes with and without calcification (Figs. 23, 24) [3].

#### Peritoneal TB

**Fig. 25:**
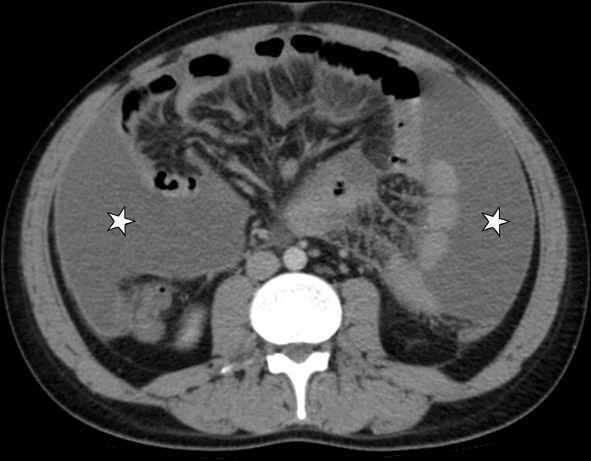
Transverse CT image with intravenous contrast of a 29-year-old male. Massive ascites is indicated in the abdomen cavity (stars). The peritoneum's thin linear contrast enhancement is also noted. Tuberculous peritonitis

**Fig. 26:**
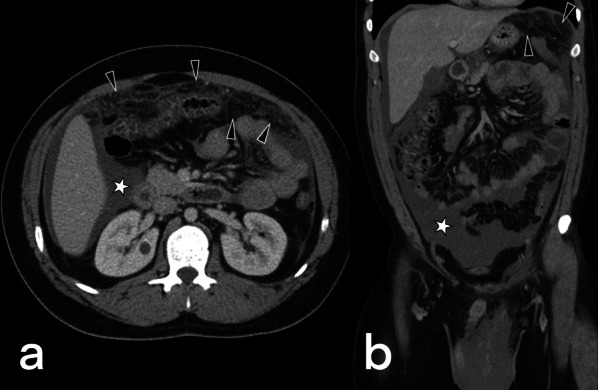
A 24-year-old male patient. Axial (**a**) and coronal postcontrast CT (**b**) images show mesenteric striation (arrowheads) and ascites (star)

**Fig. 27:**
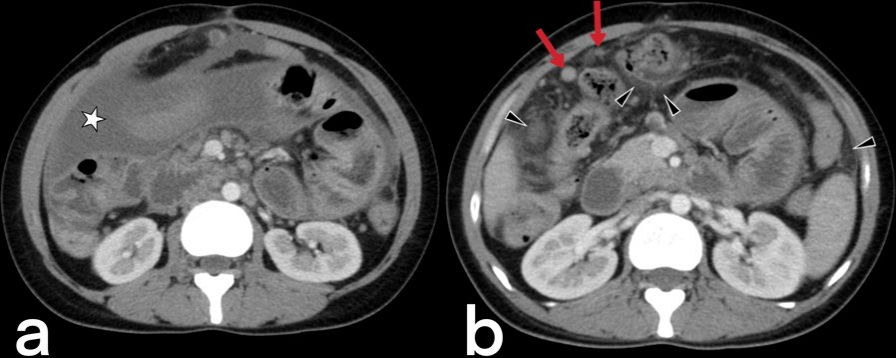
A 33-year-old female. Axial post-contrast CT images (**a**, **b**) demonstrate omental nodularity, mesenteric fat stranding (arrowheads), and ascites (star). Intraabdominal lymphadenopathies are also evident (arrows) in image **b**

This involvement has three different forms (wet, dry-plastic, and fibrotic-fixed) and is frequently associated with other abdominal TB involvements. The wet type is the most common (90%) and is observed as loculated fluid or free ascites (Fig. 25). The dry type presents the "omental cake" sign accompanied by fibrous adhesions and increased mesenteric thickness. The fibrotic type presents as a mass in the omentum or the mesentery, which can sometimes be confused with peritoneal carcinomatosis or malignancy [3]. Nevertheless, radiological findings are generally seen as a combination of these types (Figs. 26, 27) [38].

#### Hepatic and splenic TB

**Fig. 28:**
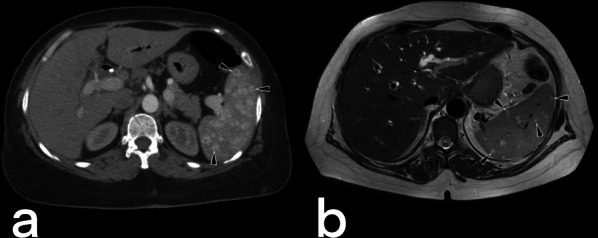
Axial contrast-enhanced CT (**a**) and axial T2 weighted MR (**b**) images of a 66-year-old female demonstrate multiple foci (arrowheads) in the spleen. Note small hypodense areas with peripheral enhancement in foci (**a**). Splenic TB

**Fig. 29:**
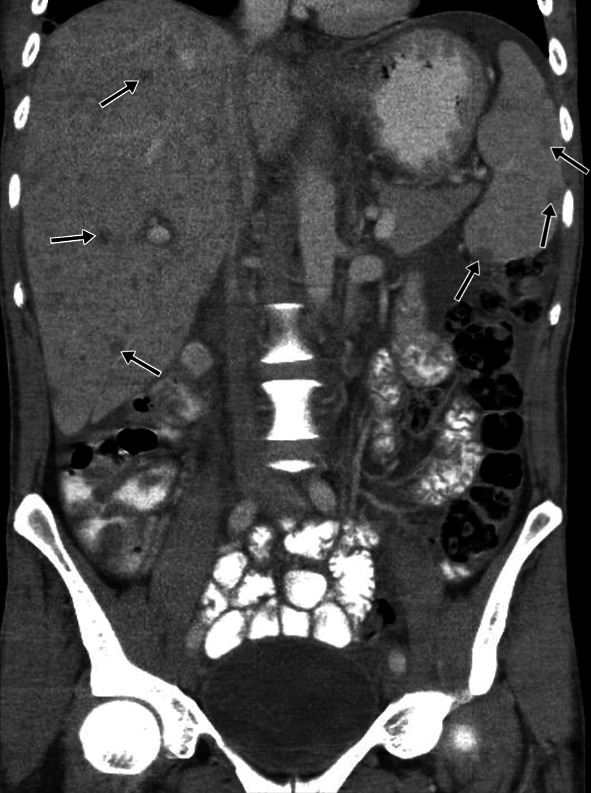
A coronal contrast-enhanced CT image of a 43-year-old male shows multiple hypodense lesions (arrows) in the liver and spleen. Hepatic and splenic TB

**Fig. 30:**
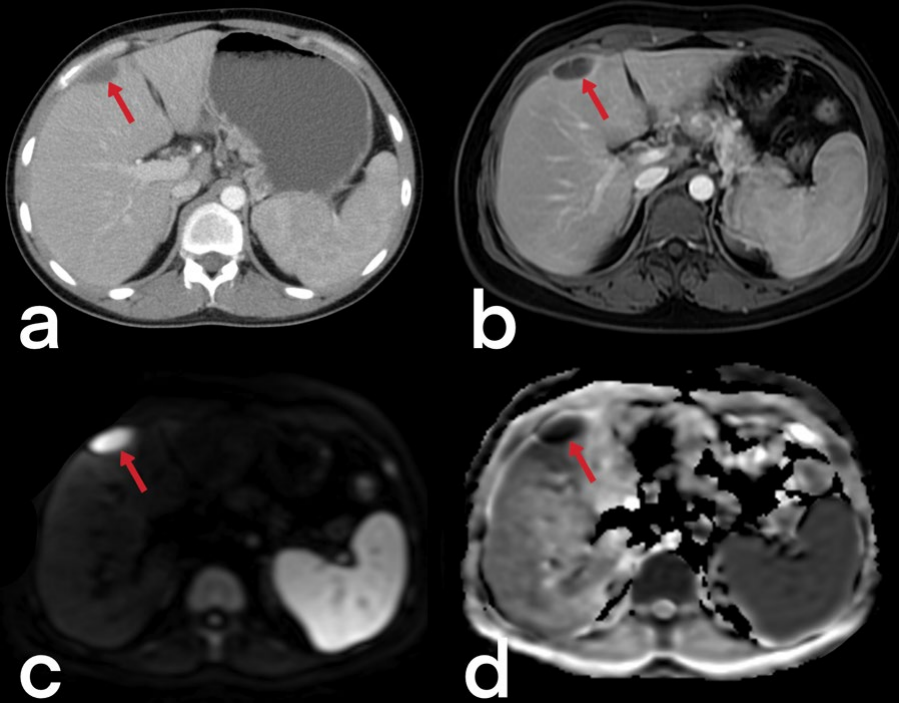
Abdominal CT scan and MR images of the same patient (in Fig. 27). Contrast-enhanced CT (**a**) and T1 weighted contrast-enhanced MR images (**b**) show a small abscess formation with rim-like enhancement in the anterior subcapsular region of the liver. Diffusion-weighted images (DWI) (**c**) and apparent diffusion coefficient (ADC) map (**d**) reveal diffusion restriction consistent with an abscess

Although hepatosplenic TB often occurs hematogenously via the hepatic artery, it may also be caused by gastrointestinal TB spreading to the liver via the portal vein [3]. Hepatosplenic TB can be seen in micronodular (the most common), macronodular (rare), and abscess (extremely rarest) forms. The micronodular form occurs due to the spread of miliary pulmonary TB. Micronodular lesions are observed on CT as hypodense, peripherally enhanced nodules with a 0.5–2 mm diameter (Figs. 28, 29). The macronodular form is rare and consists of single or multiple lesions with a diameter of 1–3 cm, with the imaging characteristics of hypointensity in the T1-weighted sequence, hypo- to hyperintensity in the T2-weighted sequence, and post-contrast peripheral and/or internal septal enhancement (honeycomb-like lesions) [3, 39–42]. Hepatic TB abscess appears as a solitary lesion filled with bacilli, usually located in the subcapsular area, with restricted diffusion on MR and peripheral enhancement on both CT and MR (Fig. 30) [36, 39, 41, 42]. Hepatosplenic TB imaging findings are not typical for TB, and a similar appearance can be detected in metastases and abscess formations [20, 36].

#### Biliary tree TB

**Fig. 31:**
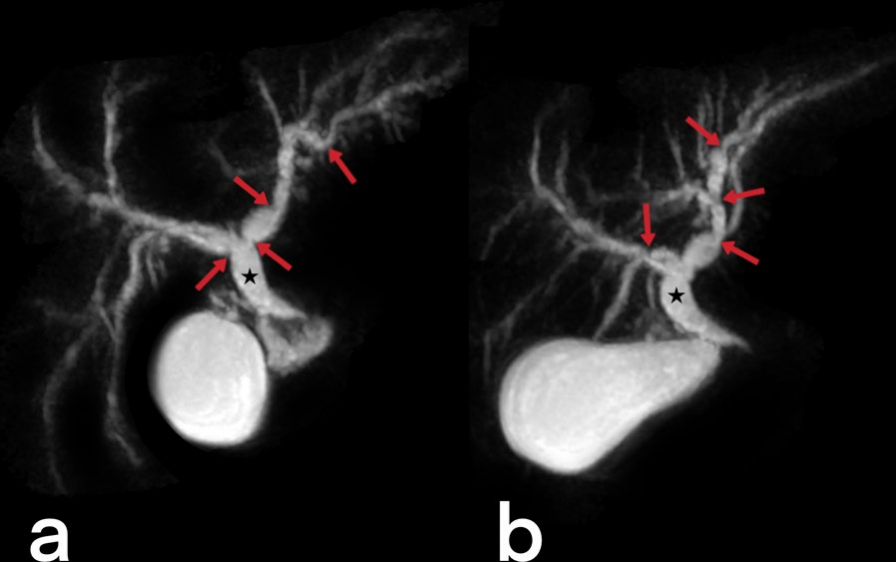
A 35-year-old male. Magnetic resonance cholangiopancreatography (MRCP) images (**a**, **b**) show extensive multifocal intrahepatic biliary strictures (arrows) and dilated common hepatic duct (star)

Biliary tract involvement is an extremely rare form of EPTB. Biliary system TB originates from via portal vein (hematogenously) or periportal an infected lymph node [36, 39, 43]. Most cases of gallbladder TB also have gallstones [43]. Generally, multiple focal strictures and post-stenotic dilatations (Fig. 31) appear in the biliary tree (on US, MRI, and MRCP) [39].

#### Pancreatic TB

**Fig. 32:**
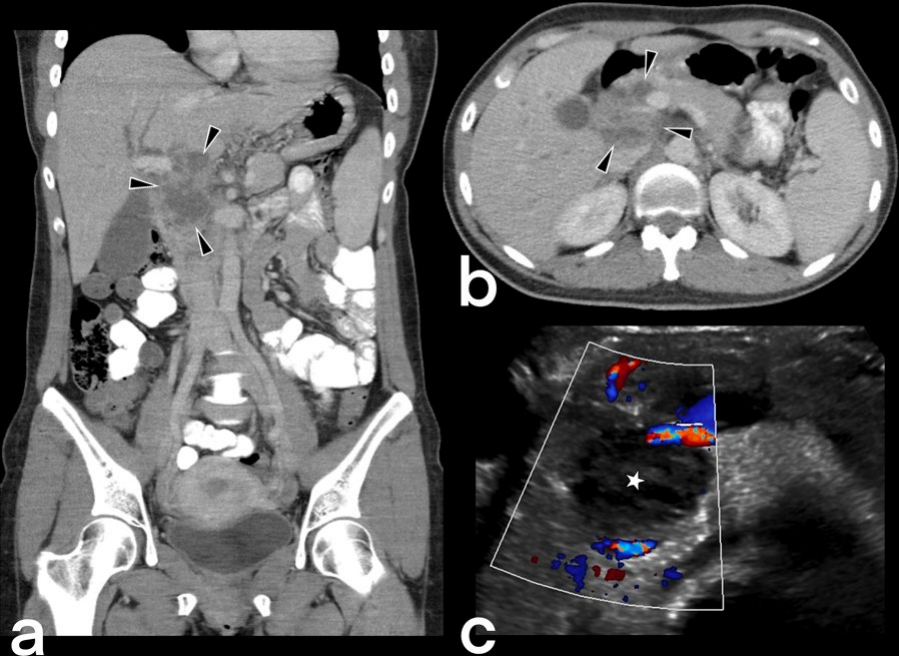
A 40-year-old female. Contrast-enhancement CT scan (**a**, **b**) and Doppler ultrasonography (**c**) demonstrated a large heterogeneous cystic-necrotic mass in the head of the pancreas

Pancreatic TB is very rare. Caseous necrosis in the pancreatic head and body is a focal, hypodense peripherally enhanced lesion with enlarged adjacent lymph nodes (Fig. 32). Neoplastic and infectious diseases are also considered in the differential diagnosis, meaning that a diagnosis is usually made based on the biopsy result [36, 42].

#### Gastrointestinal TB

**Fig. 33:**
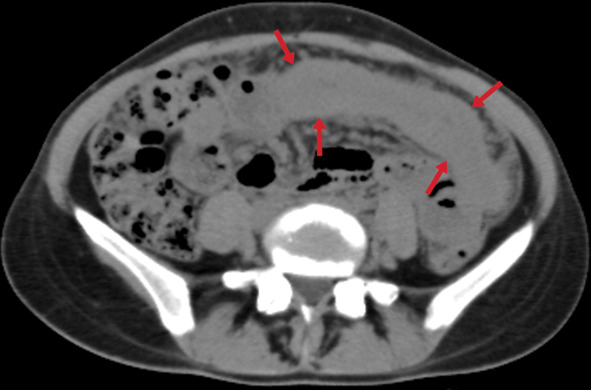
Transverse CT image without intravenous contrast of a 24-year-old female. Diffuse-symmetric wall thickening of the ileal segment is noted (arrows). Ileal TB

**Fig. 34:**
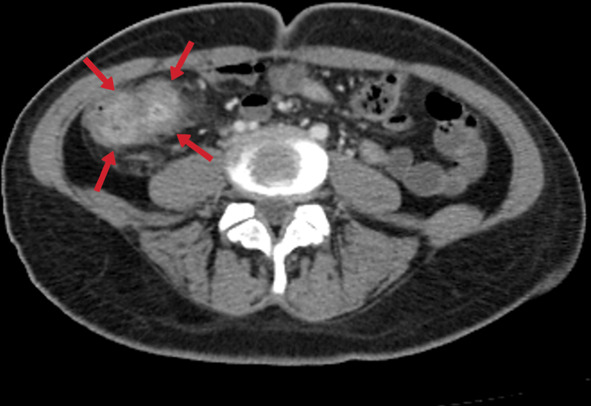
A 31-year-old female. Contrast-enhanced CT image demonstrates diffuse-symmetric wall thickening and enhancement of the cecum with surrounding inflammatory changes (arrows)

The terminal ileum is the most frequently affected area in the gastrointestinal tract (up to 90% of cases of gastrointestinal TB) due to a relative abundance of adjacent lymphoid tissue [42]. Intestinal involvement occurs in three ways: ulcerative (most common), hypertrophic, or ulcero-hypertrophic [42, 44]. CT can detect asymmetric wall thickening and mesenteric lymphadenopathy in the terminal ileum and cecum, with a thickening of the valve lips and narrowing of the terminal ileum (the Fleischner sign) also frequently observed (Figs. 33, 34). In the advanced stages of the disease, symmetrical annular strictures (napkin ring), obstruction, fibrosis, irregular contours, fistulas, sinus tracts, retraction, and pouch formation can occur [23, 40]. Complications such as obstruction, perforation, and fistula formation can develop in gastrointestinal TB, while anal fissures, fistulas, or perirectal abscesses can be seen with rectal involvement [23, 40, 42]. The differential diagnosis should also include Crohn's disease, carcinoma, lymphomatous involvement, amebiasis, *Yersinia* infection, and actinomycosis [44].

#### Adrenal TB

**Fig. 35:**
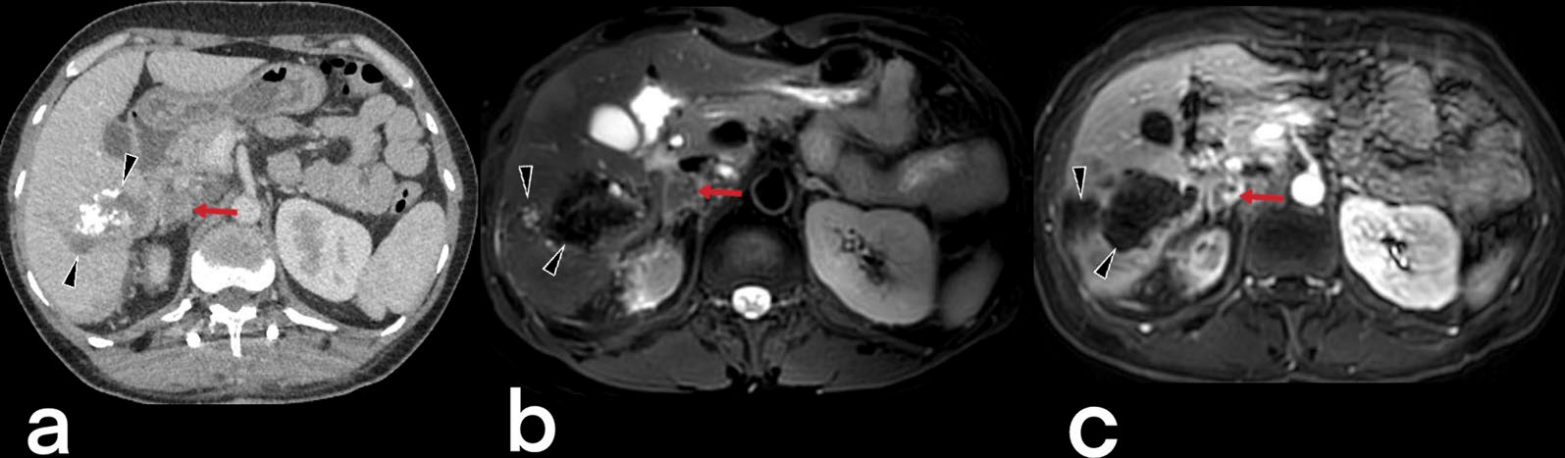
A 52-year-old female. Contrast-enhancement CT (**a**), T2 weighted MR (**b**), and contrast-enhancement T1 weighted MR (**c**) images demonstrate a heterogeneous lesion with peripheral enhancement (arrows) in the right adrenal gland (adrenal TB). Multiple calcified heterogeneous areas (arrowheads) are also seen in the liver (hepatic TB)

Adrenal gland involvement constitutes 6% of EPTB cases, with bilateral involvement often observed. Because of caseous necrosis, a patient can present clinical findings similar to Addison's disease (i.e., enlarged gland parenchyma with central hypodensity and peripheral enhancement) (Fig. 35) [3, 20, 23, 40].

### Genitourinary system

Genitourinary TB is a type of EPTB caused by the hematogenous spread of the disease. It is more common in immunosuppressed patients and older males [36]. The patient’s general condition is good and constitutional symptoms such as fever, weight loss, fatigue, and anorexia are not generally observed. The disease may be asymptomatic for a long time. Symptoms may include flank or pelvic pain, dysuria, hematuria, sterile pyuria. If female patients' genital organs are affected, menstrual irregularities, chronic lower abdominal pain, abnormal vaginal discharge, and postmenopausal bleeding may occur [4, 45, 46]. *M. tuberculosis* bacillus causes abscesses in periglomerular capillaries, but these abscesses are restricted and transformed into inactive granulomas in healthy immune systems. When immunity is suppressed, the tuberculous granulomas are reactivated and spread to adjacent papillae. They pass from the papillae to the collecting system and spread to the distal genitourinary system, causing multifocal active inflammation followed by fibrosis, scarring, and calcification [47, 48]. In genitourinary TB, lobar calcification can be detected in plain film radiography, while intravenous urography may reveal decreased calyx acuity, papillary necrosis, irregular caliectasis without pelvic dilatation, and urothelial thickening. Complications of genitourinary TB include tuberculous interstitial nephritis, sinus tracts, fistulae, and amyloidosis [49].

#### Renal TB

**Fig. 36:**
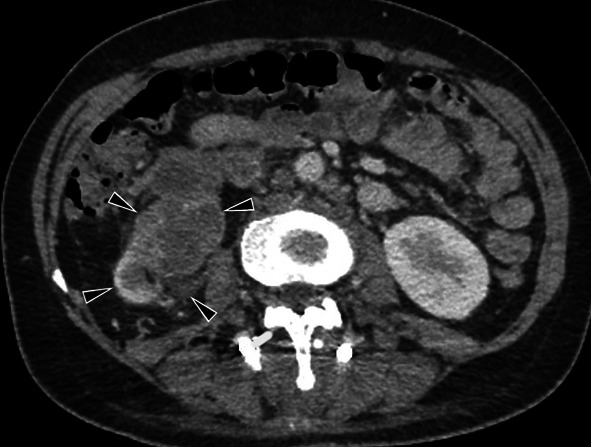
Contrast-enhancement CT scan of a 48-year-old female with renal tuberculous show debris collection within dilated calyces and parenchymal destruction (with cortical thinning)

Renal TB is generally unilateral. Intravenous urography shows a moth-eaten appearance caused by erosions in the calyx and can also show papillary necrosis, hydronephrosis with irregular contours, and contrast filling debris defects [20, 23, 40, 48]. CT can detect focal caliectasis due to infundibular strictures, renal calcification, parenchymal scarring, and hypodense parenchymal lesions (Fig. 36). In end-stage TB (auto-nephrectomy), calcifications with lobar distribution are pathognomonic [20].

#### Ureteral TB

**Fig. 37:**
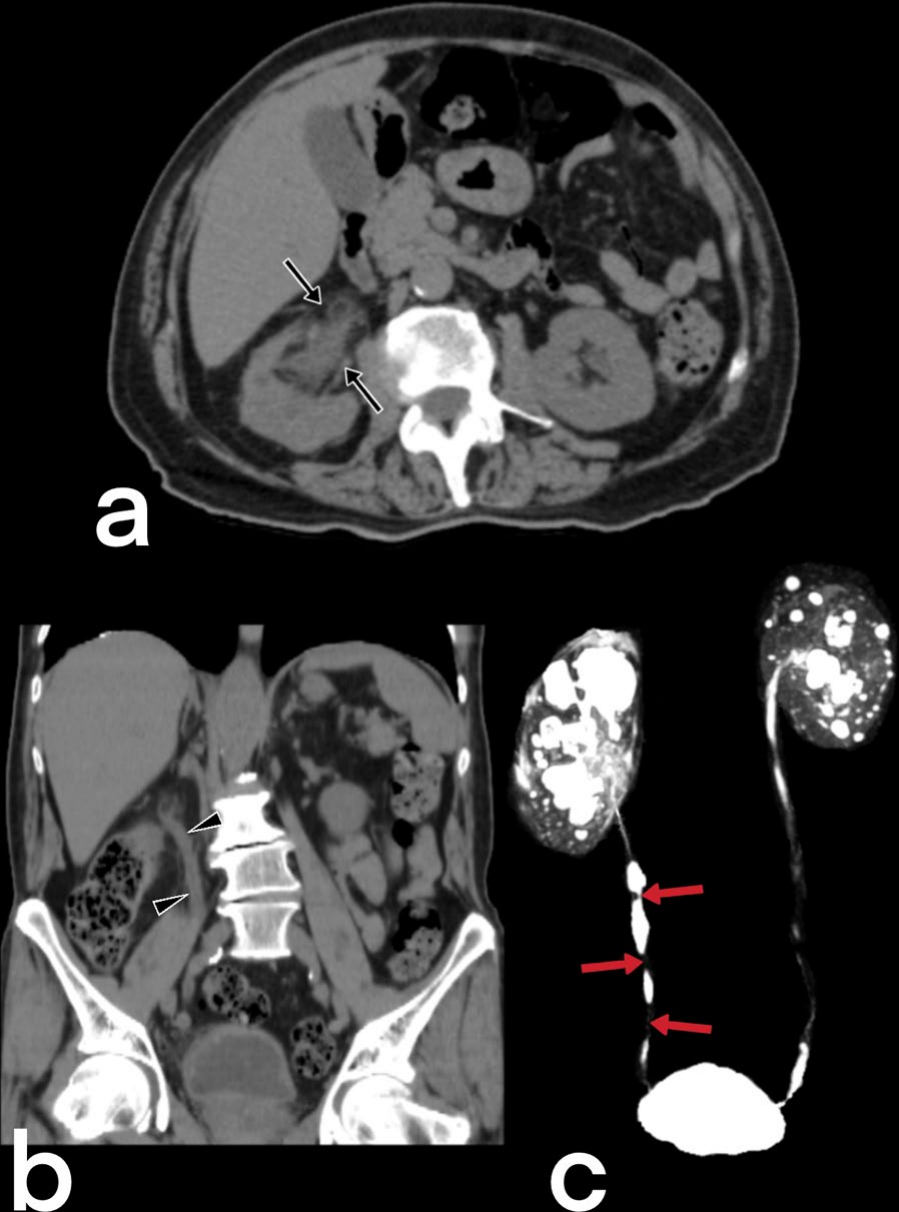
Axial (**a**) and coronal (**b**) non-contrast CT scan of a 74-year-old male with urinary tuberculous reveal debris collection within the dilated right renal pelvis and dilated right ureter with diffuse wall thickening. 3D MIP Coronal T2-weighted MR urogram shows strictures (arrows) of the right ureter

Thickening of the ureter wall is observed in acute TB involvement, while strictures in the lumen and a shortening of the ureter are detected in chronic TB involvement (Fig. 37) [20, 23, 40, 48].

#### Urinary bladder TB

**Fig. 38:**
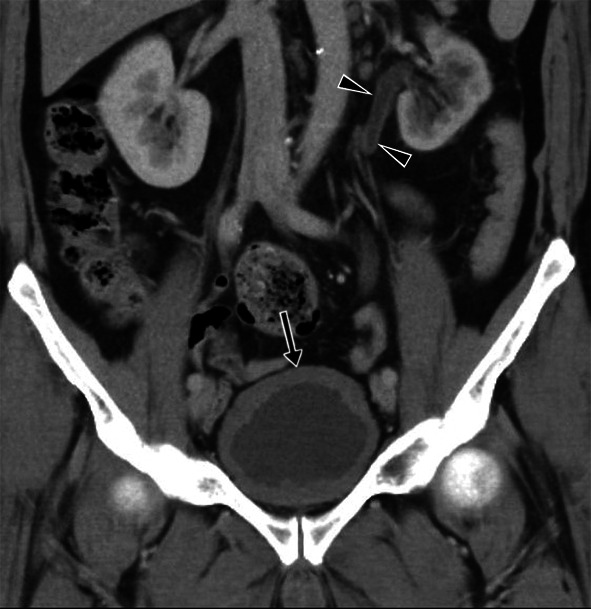
A 47-year-old male. Coronal post-contrast CT scan shows diffuse thickening of the bladder wall (arrow) and dilated left proximal ureter (arrowheads). Urinary TB

Urinary bladder involvement is caused by the spread of the infection from the kidneys. Bladder wall thickening, ulceration, and filling defects due to granulomatous material are observed in acute TB (Fig. 38), while decreased bladder capacity and irregular and calcified appearances in bladder contours indicate chronic TB [20, 23, 40]. Fibrotic changes can also cause hydroureteronephrosis [20].

#### Involvement of the genital organs

**Fig. 39:**
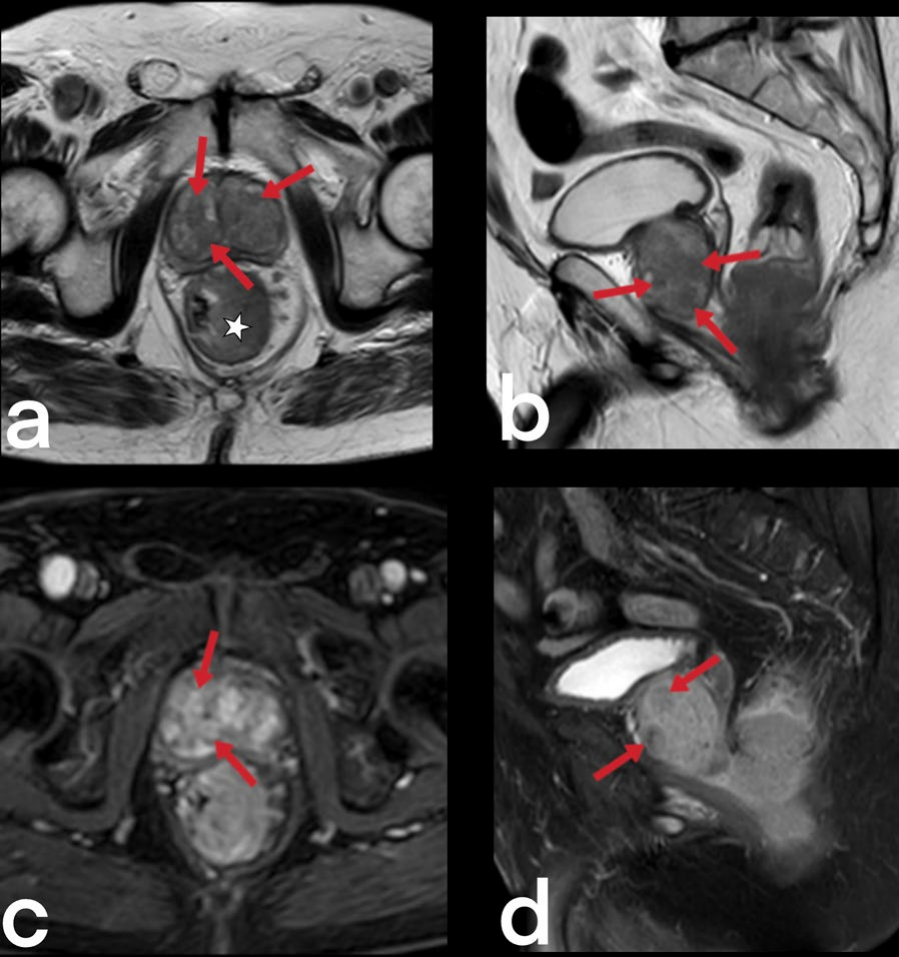
Tuberculous prostatitis (abscesses) in a 53-year-old male. T2 weighted axial (**a**) and sagittal (**b**) MR images demonstrate focal, hypointense lesions within the prostate gland (arrows). Contrast-enhancement T1 weighted axial (**c**) and sagittal (**b**) MR images show heterogeneous lesions with peripheral enhancement (arrows). A rectal tumor (star) is also seen

**Fig. 40:**
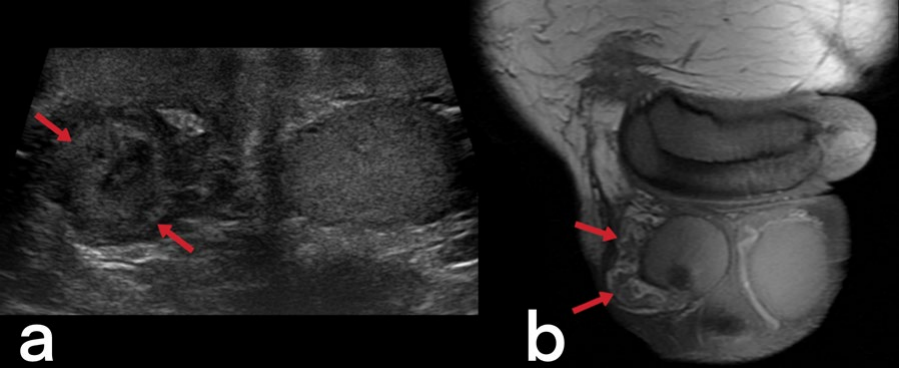
A 38-year-old male. Scrotal US image (**a**) and post-contrast coronal T1 weighted MR image (**b**) reveal diffuse heterogeneity, deformation, and dilated cystic lump with peripheral enhancement in the right epididymis (arrows)

**Fig. 41:**
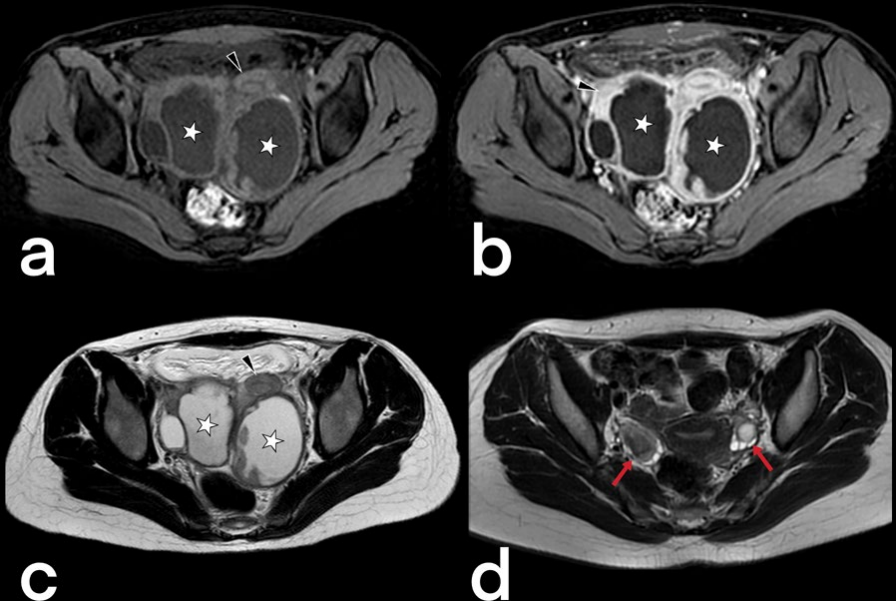
Bilateral tubo-ovarian TB in a 15-year-old-female with pelvic pain. Axial pre-contrast T1 weighted (**a**), post-contrast T1 weighted (**b**), and T2 weighted (**c**) MR images show bilaterally enlarged ovary (stars) and dilated fallopian tubes (arrowheads). On T2-weighted MR image obtained after antituberculous therapy of 10 months, bilateral ovaries (arrows) and fallopian tubes (not shown) appear normal (**d**)

**Fig. 42:**
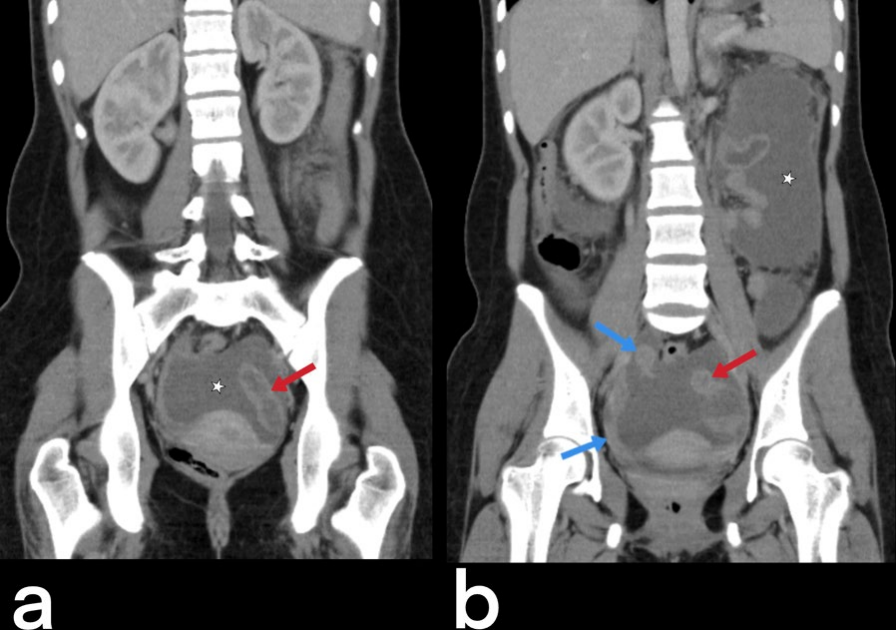
Bilaterally hydrosalpinx (right: blue arrows, left: red arrow) and ascites (stars) in the peritoneal cavity are observed on coronal contrast-enhancement CT images (**a, b**) of the same patient (in Fig. 41)

**Fig. 43:**
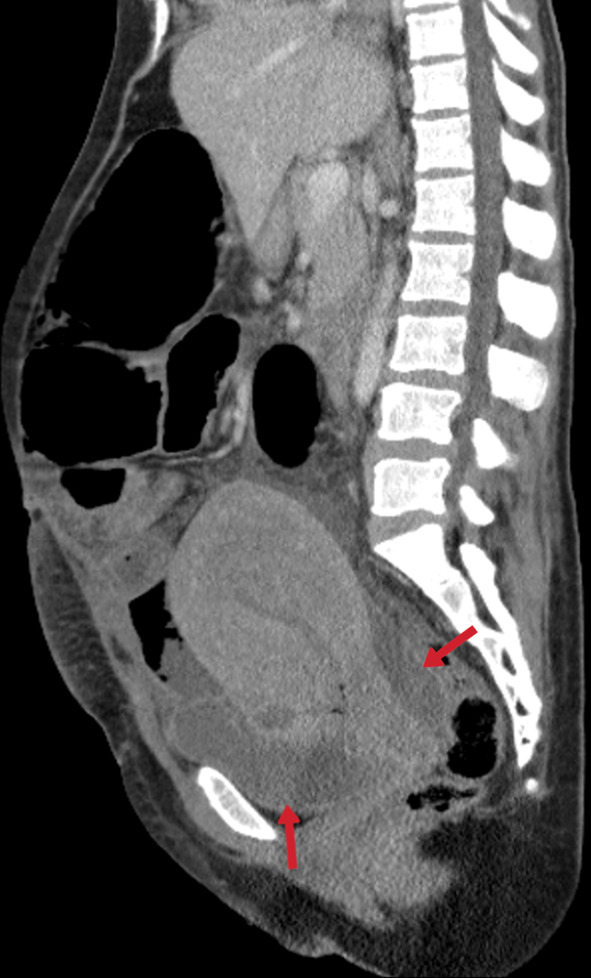
Sagittal-reformatted abdomen CT image of a 22-year-old female with lower abdominal pain and fever obtained after C/S surgery. The uterus is enlarged due to prior pregnancy. Free fluid is noted in the pelvis (arrows). Tuberculous pelvic inflammatory disease

Genital TB affects both men and women and can cause infertility [23, 50]. Involvement of the prostate, seminal vesicles, epididymis, and testicles can be seen in men, while women can present with ovary, fallopian tube, and endometrium involvement. Primary genital TB can develop in women’s cervix, vagina, or vulva. The transmission route can be through infected semen from male partners with active genitourinary TB or through the sputum used as a sexual lubricant of their partners with pulmonary TB [23]. Imaging findings can include prostatitis (linear, low-signal areas in the peripheral zone of the prostate in T2-weighted MRI images; prostate cancer is also considered in the differential diagnosis) (Fig. 39), prostate abscesses (restricted diffusion finding in MRI), epididymo-orchitis (dimensional increase and heterogeneity in the epididymis and testicles) (Fig. 40), and calcifications in the prostate and seminal vesicles [23, 40]. In women, salpingitis, hydrosalpinx, tubo-ovarian abscesses (Figs. 41, 42), synechiae in the fallopian tubes, ascites in the pelvic peritoneal cavity (Fig. 43), and adhesions in the endometrium resulting from TB may cause infertility [23, 48]. Differential diagnosis includes ectopic pregnancy, pelvic inflammatory disease, endometriosis, ovarian malignancy, endometrial carcinoma, and dysfunctional uterine bleeding [46].

### Musculoskeletal system

Musculoskeletal TB is seen in 1–3% of all TB cases and is usually caused by the hematogenous spread of TB or after trauma. Musculoskeletal TB is grouped as tuberculous spondylitis (Potts' spine), peripheral tuberculous arthritis, osteomyelitis, and soft tissue TB, including tenosynovitis, abscesses, and bursitis [3].

#### Tuberculous spondylitis

**Fig. 44:**
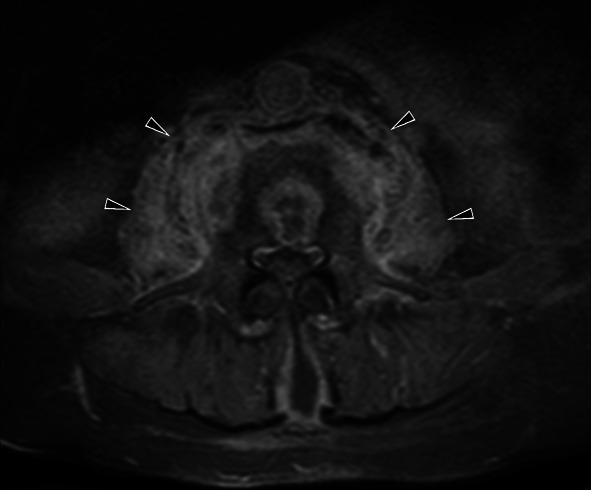
Contrast-enhanced T1 weighted axial MR image of a 52-year-old male appears an inflammatory mass lesion within the adjacent soft tissues to the body of the fourth lumbar vertebra be caused by a phlegmon and abscess

**Fig. 45:**
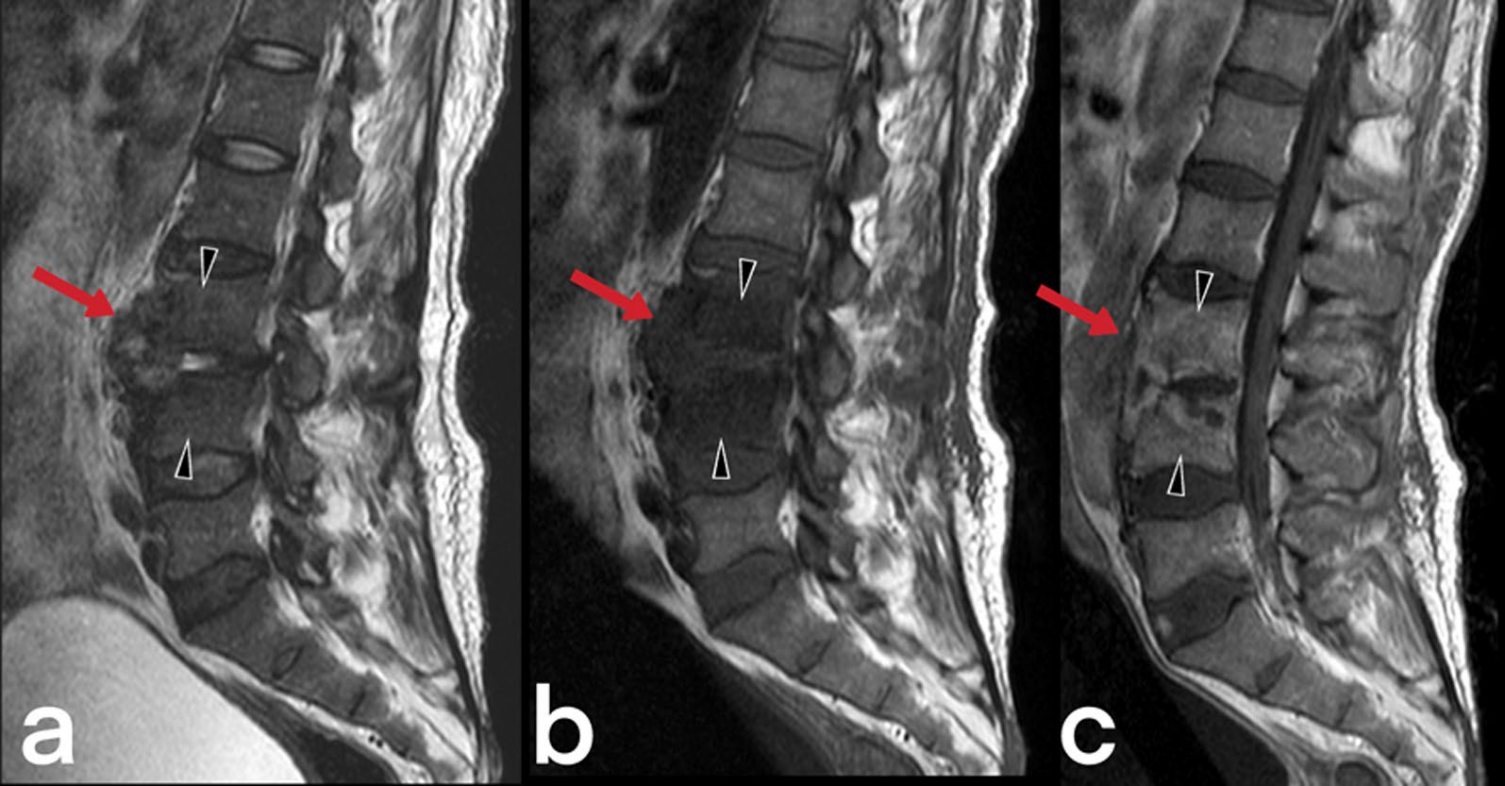
MR images of lumbar vertebrae of a 47-year-old male with tuberculous spondylodiscitis. Sagittal T2-weighted image (**a**) shows the heterogeneous low signal in the anterior parts of the L3 and L4 vertebral bodies and L3-4 intervertebral disc, which offers minimal anterior subligamentous extension (arrows). Pre-contrast (**a**) and post-contrast (**b**) T1 weighted images reveal diffuse contrast enhancement of both vertebral bodies in keeping with spondylodiscitis (arrowheads)

**Fig. 46:**
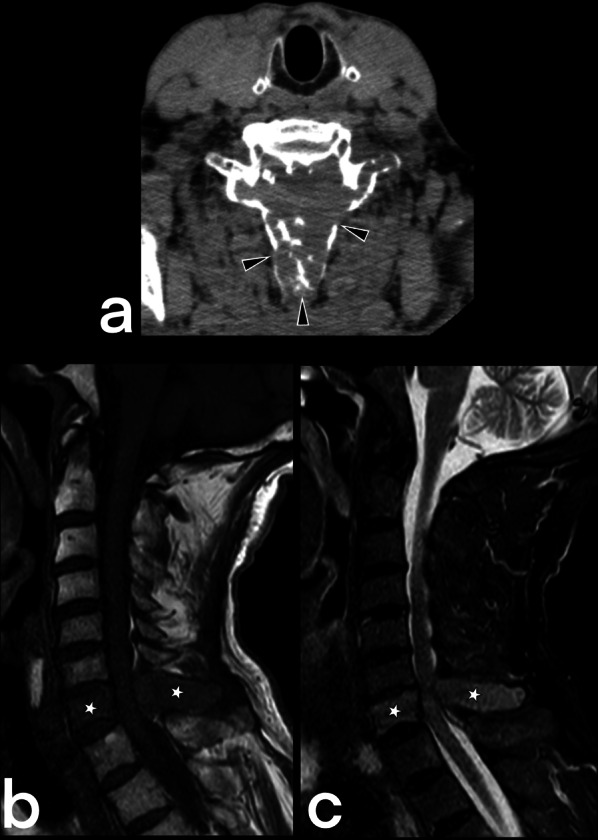
A 41-year-old male with pain in the posterior neck region. Non-contrast axial CT image (**a**) demonstrates destruction and bone erosion of the posterior spinous process of the C7 vertebra (arrowheads). Pre-contrast sagittal T1 weighted image (**b**) reveals diffuse decreased signal intensity within the c7 vertebral body and posterior spinous process. The lesion shows prominent diffuse contrast enhancement on sagittal (**c**) T1-weighted images (stars)

The spine is the most common bony TB involvement site, with the lower thoracic and upper lumbar vertebrae being most frequently affected. Vertebral body involvement adjacent to the disc, subligamentous involvement, and spread to the superior or inferior vertebrae without any effect on the vertebral disc are observed [3, 40]. MRI is helpful in early-stage diagnosis, with typical radiological findings showing hypointensity on T1-weighted images, hyperintensity on T2-weighted images, and post-contrast irregular, patchy enhancement in the anterior part of the vertebra corpus end platelets indicating bone marrow edema (Fig. 44). Subligamentous spread, a paraspinal localization, a smoothly circumscribed abscess containing a small amount of edema around three or more vertebrae (cold abscess), less bone destruction despite significant paraspinal involvement, and relative preservation of the intervertebral disc are all significant indications of tuberculous spondylodiscitis (Figs. 45, 46) [3, 23]. In untreated cases, vertebral collapse and anterior angulation can cause kyphosis, the Gibbus deformity [20].

The extravertebral involvement of TB is rare (1–2%), with peripheral arthritis, osteomyelitis, tenosynovitis, and bursitis observed in the order of decreasing frequency [23]. The disease spreads through the joint space through hematogenous subsynovial vessels or the epiphysis in adults and the metaphysis in children. This involvement manifests as trabecular and cortical destruction, periosteal reaction, and a soft tissue mass [23, 40]. Other pyogenic infections (such as brucellosis, fungal infection), metastatic tumors, and sarcoidosis should be considered in the differential diagnosis. Pyogenic infections are more aggressive, and intervertebral disc destruction is in the foreground. Paravertebral soft tissue mass is more common in TB spondylodiscitis [40].

#### Tuberculous arthritis

**Fig. 47:**
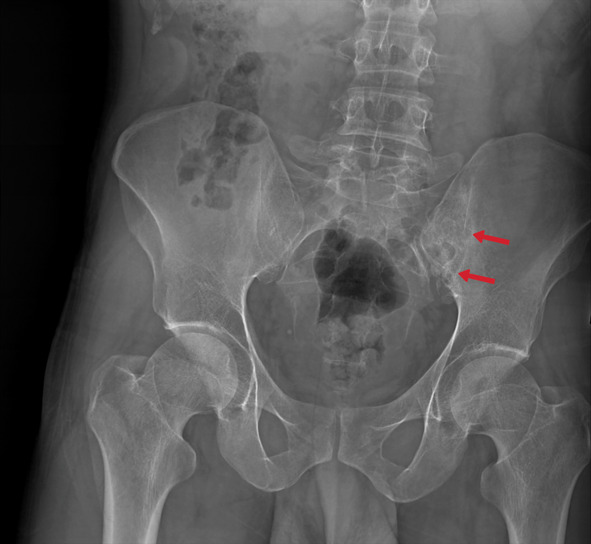
Pelvic x-ray of a 46-year-old male. The late finding of tuberculous sacroiliitis is characterized by degenerative changes in the left sacroiliac joint (arrows)

**Fig. 48:**
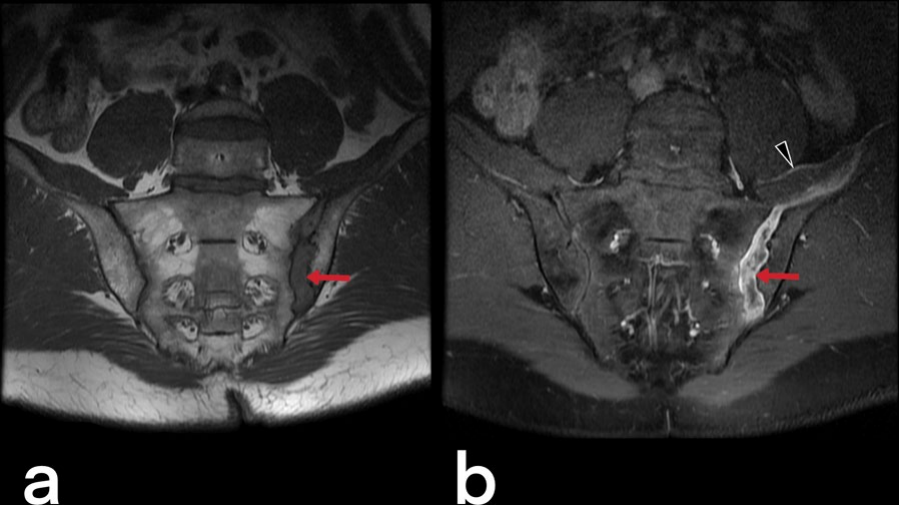
MR images of a 43-year-old male. Pre-contrast T1 weighted (**a**) and post-contrast (**b**) T1 weighted images demonstrate contrast enhancement in the synovium of the left sacroiliac joint in keeping with acute sacroiliitis (arrows). Arrowhead shows an abscess formation in the left iliac muscle that enhances peripherally after contrast injection

**Fig. 49:**
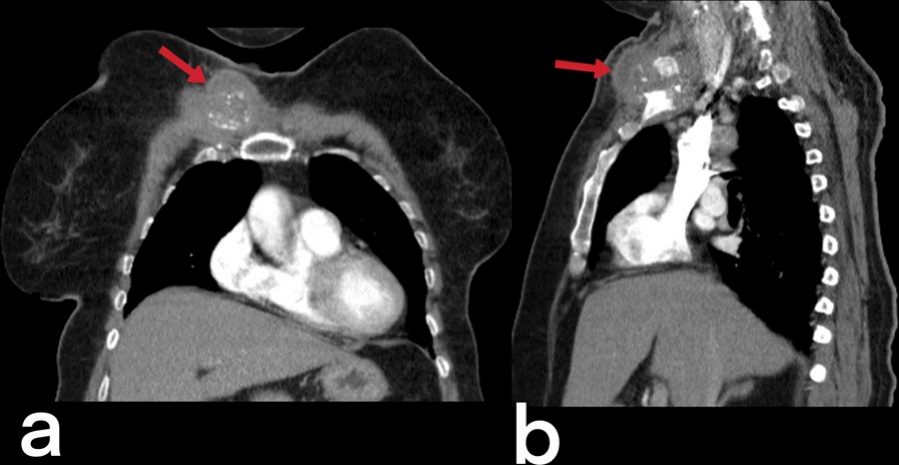
Coronal (**a**) and sagittal (**b**) reformatted CT images of a 70-year-old female who has a complaint of swelling in the anterior wall of the chest. Both images reveal mass-like lesions in the right sternoclavicular joint, causing bone destruction containing small calcified areas. The patient was diagnosed with TB after the biopsy

**Fig. 50:**
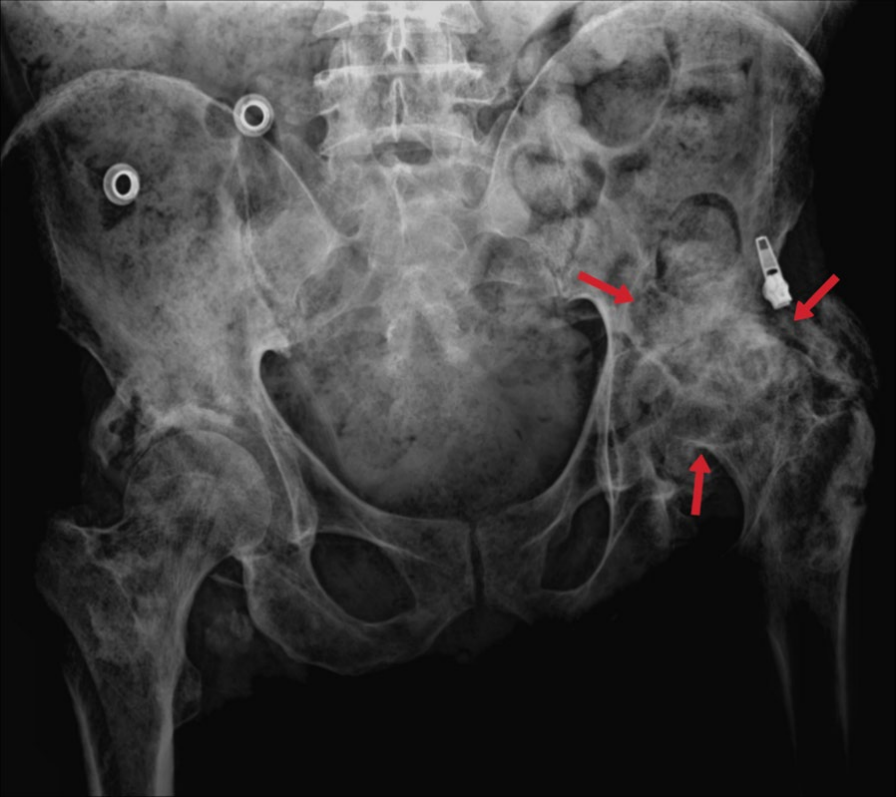
Pelvic X-ray of a 53-year-old female. The left hip joint sequela of TB is characterized by degenerative arthritis (arrows)

Tuberculous arthritis is mainly observed in weight-bearing joints such as the sacroiliac (Figs. 47, 48), hips, and knees and involves a single joint at a rate of 90%. Radiographic findings include periarticular/juxta-articular osteoporosis, peripherally located marginal bone erosion, and progressive joint space narrowing, known as the triad of Phemister (Figs. 49, 50). Ankylosis and degeneration are also observed later [3, 23].

Although there is significant synovitis in early-stage TB arthritis, the articular cartilage remains intact [23]. Periarticular soft tissue calcification and abscesses can also be observed [23, 51].

#### Tuberculous osteomyelitis

**Fig. 51:**
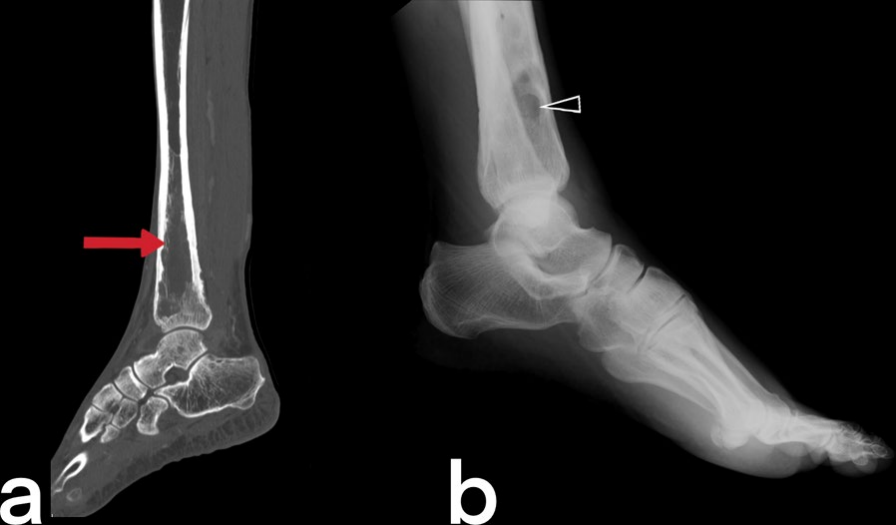
CT (**a**) and lateral ankle direct X-ray (**b**) images of two different patients. Lytic areas are observed in the tibia in both cases with proven tuberculous osteomyelitis and abscess

**Fig. 52:**
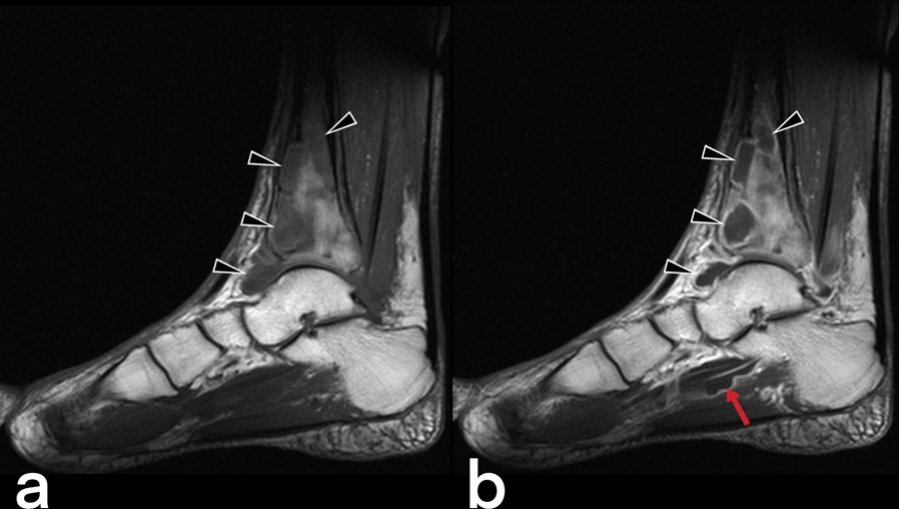
MR images of a 59-year-old male. Pre-contrast (**a**) and postcontrast (**b**) T1 weighted images demonstrate intraosseous (arrowheads) and soft tissue abscesses (arrow) in the distal part of the tibia and plantar face of the foot

**Fig. 53:**
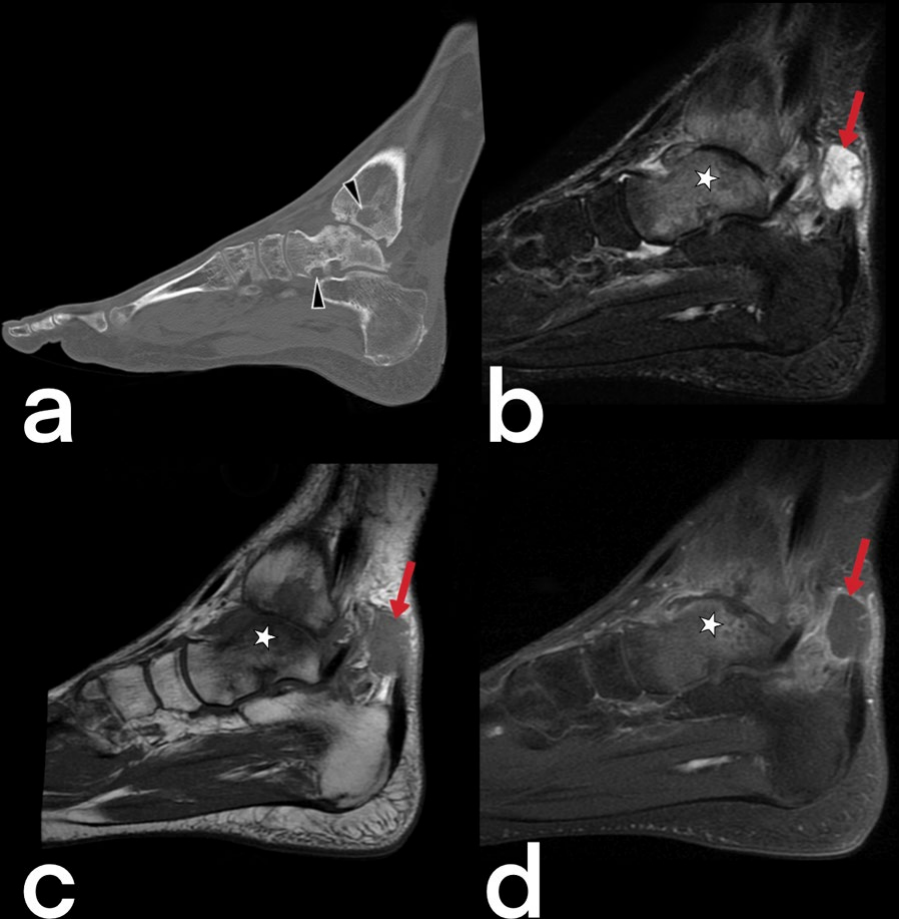
Sagittal reformatted CT (**a**) and MR images (**b**–**d**) of a 48-year-old male. CT image shows focal low-attenuation areas in the distal part of the tibia in keeping with abscesses and bone destructions (arrowheads). On sagittal T2-weighted (**b**), pre-contrast T1-weighted (**c**), and post-contrast T1 weighted (**d**) MR images reveal extensive signal intensity change within the bone marrow of the talus and distal tibial part (stars). MR images also demonstrate an abscess in the adjacent soft tissue to the Achilles tendon (arrows)

**Fig. 54:**
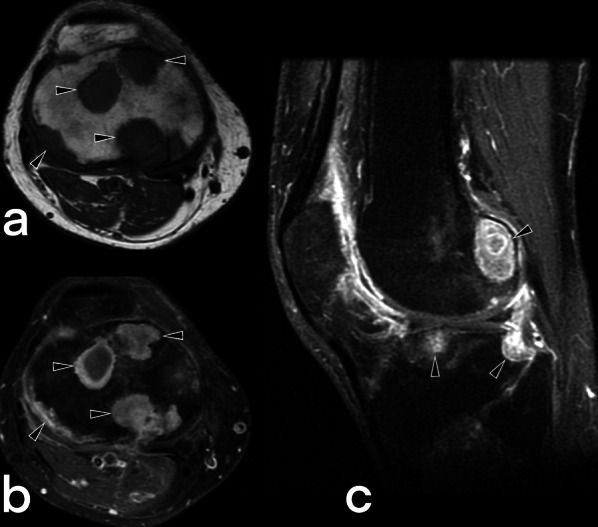
MR images of a 60-year-old male. Pre-contrast axial (**a**), post-contrast axial (**b**), and sagittal (**c**) T1 weighted images reveal intraosseous abscesses (arrowheads) with peripheral enhancement in the distal part of the femur and proximal tibia

Tuberculous osteomyelitis affects the femur, tibia, bones of hands and feet [51, 52]. Plain film X-ray shows osteopenia in the metaphysis surrounded by lytic lesions with minimal sclerosis (Fig. 51) [53]. MRI imaging findings are variable appearance similar to pyogenic osteomyelitis (low T1, high T2) and revealed earlier than CT and X-ray [54]. Marrow edema (high T2), intraosseous abscesses (T2 hyperintense center (caseous center) and peripheral enhancement on T1), abnormalities of adjacent muscles and extraosseous soft tissues (such as edema, abscess, fistula) may be seen (Fig. 52, 53, 54) [54, 55]. The spread of infection to the epiphysis in children is a typical feature that helps distinguish TB from pyogenic infection [20]. In children, fusiform soft tissue swelling in the short tubular bones of the hands and feet that may accompany periostitis is consistent with tuberculous dactylitis [3, 40, 51]. A balloon-like swelling and a cyst-like cavity appearance in the involved bone are known as "spina ventosa" (a wind-filled sail) [40, 56].

#### Muscle, skin and subcutaneous fat tissue TB

**Fig. 55:**
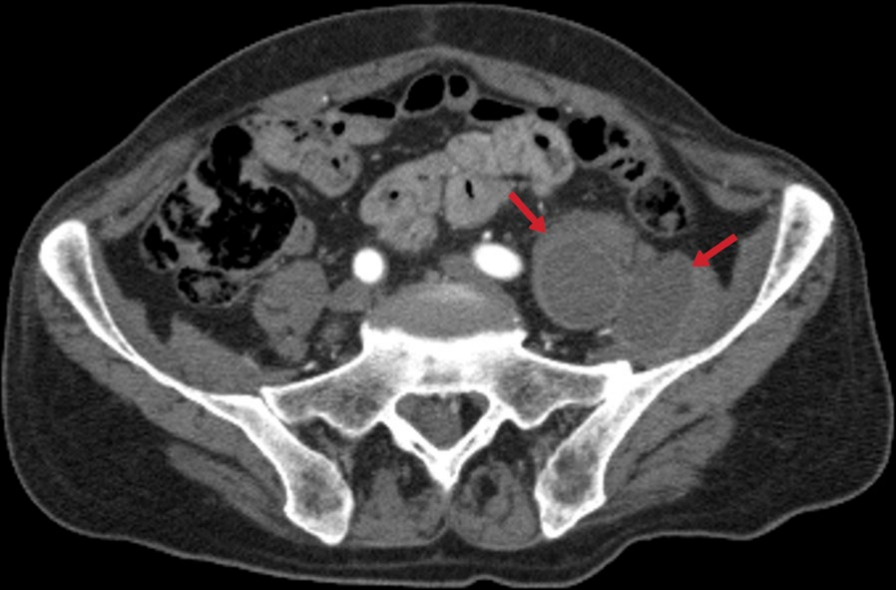
A contrast-enhanced CT image of a 69-year-old male demonstrates the left iliopsoas abscess (arrows)

**Fig. 56:**
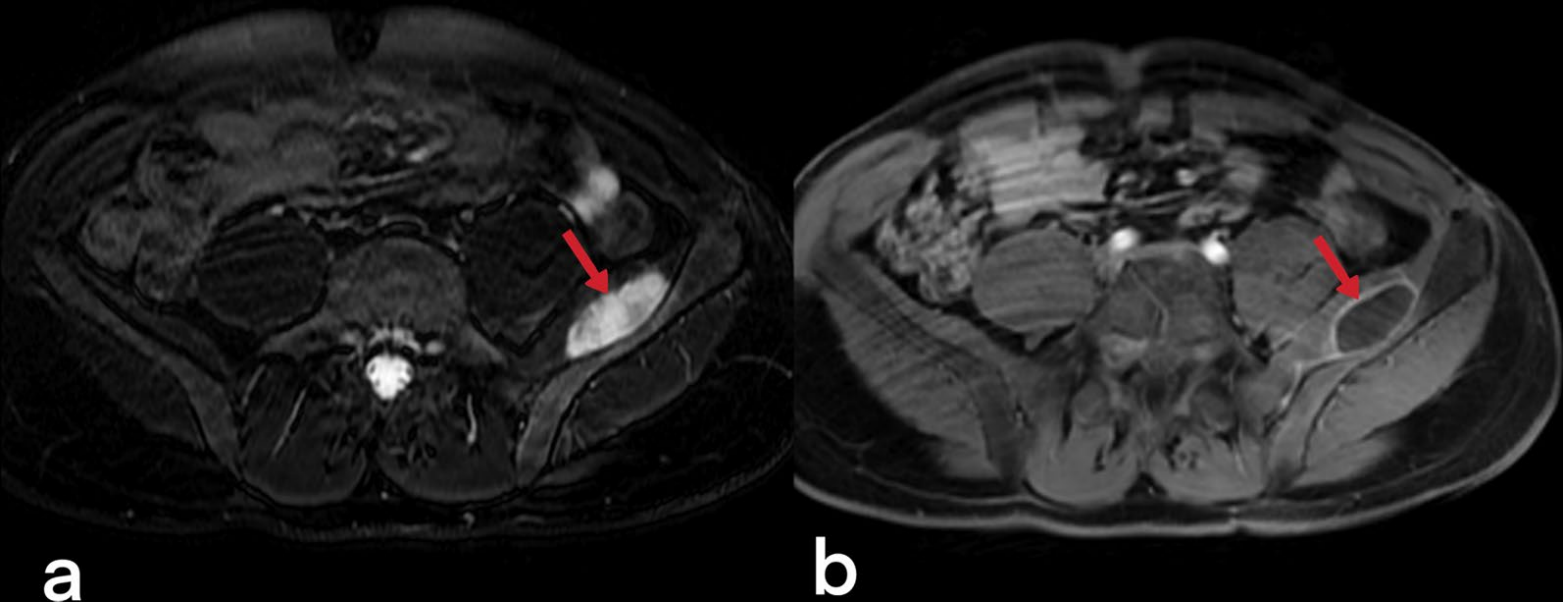
T2 weighted (**a**) and post-contrast T1 weighted MR images of a 35-year-old male demonstrate abscess formation with peripheral enhancement in the left iliac muscle (**b**)

Muscle, skin, and subcutaneous adipose tissue involvements are very rare [52]. In patients with tuberculous arthritis/osteomyelitis, the disease can spread through ruptured necrotic lymph nodes or from adjacent foci [51, 52]. Psoas abscesses (Fig. 55) usually develop secondary to spondylodiscitis [53]. Notable edema, myositis, or cellulitis is not observed around the lesion in muscle involvement (Fig. 56) [23]. Skin TB is seen as a result of exogenous inoculation, spreading from the adjacent foci of infection or through the hematogenous route. Cutaneous TB presents in different forms and can be seen as patches, plaque, macules, papules, nodules, abscesses, erosions, and ulcers [57].

### Treatment and follow-up

There are no medical treatment guidelines specific to EPTB. As in pulmonary TB treatment, EPTB is treated by 6–18 months of antitubercular chemotherapy. In tuberculous meningitis, corticosteroids are often used in addition to antitubercular chemotherapy, which can also help prevent fibrotic sequelae, including constrictive pericarditis and stenosis that may develop in hollow internal organs such as the intestine and ureter [7, 58].

In a patient that recovers from the disease after treatment, TB lesions may reduce clinically or radiologically, or new lesions may develop a phenomenon known as a paradoxical reaction. Paradoxical reactions occur more frequently in EPTB than in pulmonary TB; lymph nodes, the pleura, and the CNS are the most frequently affected areas [58].

Surgery (such as lumpectomy or mastectomy in breast tuberculosis, surgical repair of kyphosis sequela in spinal tuberculosis, a non-functioning kidney nephrectomy, or placement of a ventriculoperitoneal shunt for the treatment of hydrocephalus) is an option when there is no response to medical treatment. Abscesses can be treated effectively with US or CT-guided external drainage [7, 58].

For patients with EPTB, there is usually no need for a second biopsy or bacteriological evaluation to assess the response to treatment. The response should generally be evaluated based on clinical and radiological findings [7, 58].

## Conclusion

TB is a severe disease that can affect almost every organ and tissue of the body and should be considered in the differential diagnosis, particularly in patients with immune system disorders. Some radiological involvement patterns in EPTB are specific to the disease and can assist in early diagnosis. Knowing radiological findings can prevent unnecessary biopsy (such as brain biopsy) and surgical procedures that may result in organ loss (such as mastectomy, oophorectomy, splenectomy). Early diagnosis and treatment are the most critical factors in reducing morbidity associated with the disease.

## Data Availability

Not applicable.

## Abbreviations

CNS: Central nervous system
CT: Computed tomography
EPTB: Extrapulmonary tuberculosis
MRI: Magnetic resonance imaging
TB: Tuberculosis
